# Design, synthesis and evaluation of a tripodal receptor for phosphatidylinositol phosphates

**DOI:** 10.1038/s41598-020-75484-w

**Published:** 2020-10-28

**Authors:** Katharina Reeh, Peter A. Summers, Ian R. Gould, Rudiger Woscholski, Ramon Vilar

**Affiliations:** 1 Department of Chemistry, Imperial College London, White City Campus, 84 Wood Lane, London, W12 0BZ UK; 2 Institute of Chemical Biology, Imperial College London, White City Campus, 84 Wood Lane, London, W12 0BZ UK

**Keywords:** Chemistry, Chemical biology, Organic chemistry, Supramolecular chemistry

## Abstract

Phosphatidylinositol phosphates (PIPs) are membrane phospholipids that play crucial roles in a wide range of cellular processes. Their function is dictated by the number and positions of the phosphate groups in the inositol ring (with seven different PIPs being active in the cell). Therefore, there is significant interest in developing small-molecule receptors that can bind selectively to these species and in doing so affect their cellular function or be the basis for molecular probes. However, to date there are very few examples of such molecular receptors. Towards this aim, herein we report a novel tripodal molecule that acts as receptor for mono- and bis-phosphorylated PIPs in a cell free environment. To assess their affinity to PIPs we have developed a new cell free assay based on the ability of the receptor to prevent alkaline phosphatase from hydrolysing these substrates. The new receptor displays selectivity towards two out of the seven PIPs, namely PI(3)P and PI(3,4)P_2_. To rationalise these results, a DFT computational study was performed which corroborated the experimental results and provided insight into the host–guest binding mode.

## Introduction

**Figure 1:**
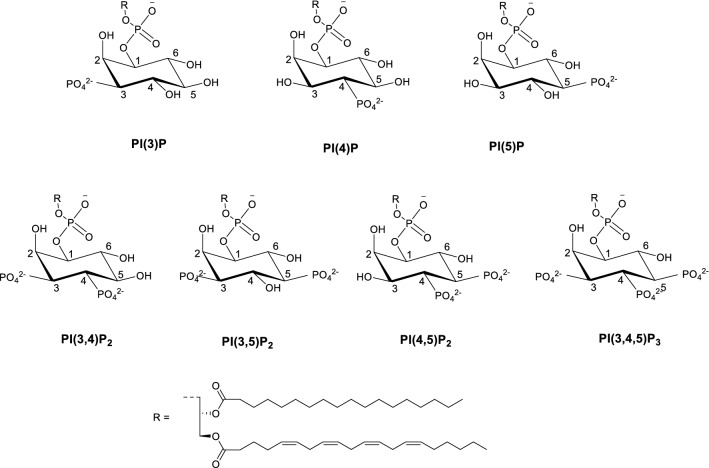
Chemical structure of the seven biologically relevant phosphatidylinositol phosphates.

Phosphatidylinositol phosphates (PIPs) are membrane phospholipids that play crucial roles in a wide range of cellular functions^1^. There are seven biologically relevant PIPs all of which are derived from D-*myo*-phosphatidylinositol (PI—see Fig. 1)^2^. Reversible phosphorylation of the hydroxyl groups at the D3, D4 or D5 position of this inositol headgroup generates seven different combinations of phosphatidylinositols (PIs), including three monophosphorylated (PI(3)P, PI(4)P and PI(5)P), three biphosphorylated (PI(3,4)P_2_, PI(4,5)P_2_ and PI(3,5)P_2_) and one triphosphorylated (PI(3,4,5)P_3_) PI species^3^. Changes of cellular PIP levels and disruption of the equilibrium between the different PIPs have been linked to the development of a host of diseases, such as Alzheimer’s and cancer^4, 5^.

Proteins are able to recognise different PIPs via a variety of specific domains^6^. This drives the recruitment of proteins to specific intracellular compartments where the respective domains then serve as a membrane tether for the rest of the protein. The Pleckstrin Homology (PH) domain forms the largest group of recognition modules^7^. It is composed of approximately 120 amino acid residues and is present in over 270 human proteins which have been associated with a variety of processes such as intracellular signalling, membrane trafficking, cytoskeletal rearrangements and lipid metabolism. A subset of PH-domains display high affinity and specificity towards individual PIPs, primarily towards PI(3,4)P_2_, PI(4,5)P_2_ and PI(3,4,5)P_3_. The FYVE domain is another important recognition motif for PIPs; it is a zinc finger which contains approximately 70 residues and has been identified in 28 different human proteins^8^. The three distinguishing motifs of this domain are the conserved sequences based on WxxD, RR/KHHCR and RVC motifs, which define the positively charged binding site for PI(3)P. The FYVE domain has nanomolar affinity for PI(3)P and is critical for the catalytic activity of certain enzymes as well as their translocation to endosomal membranes.

Due to their high affinity and selectivity, these domains have been employed as tools to study and visualize PIPs in cellular processes. However, their large size (as compared to the PIPs) makes them impractical for some applications. Therefore, there is interest in the development of small molecules with high affinity and selectivity for specific PIPs. Such molecules would be ideal tools to study PIPs in cells and, potentially, could disrupt their metabolism and hence be the basis for drug development.

**Figure 2:**
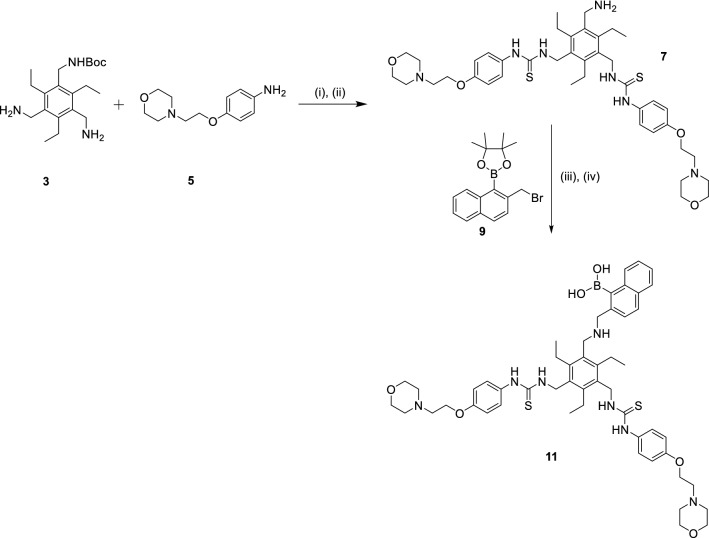
Reaction scheme for the synthesis of tripodal receptor **11**. (1) TDCI, MeCN, at 0 °C for 20 min followed by 12 h at 80 °C; (2) TFA, CH_2_Cl_2_, RT, 12 h; (3) K_2_CO_3_, DMF, RT, 5 h; (4) TFA, H_2_O, 70 °C, 12 h.

Anslyn^9, 10^, Ahn^11^, Yoon^12, 13^, Aoki^14, 15^ and Best^16^ have previously reported receptors for inositol phosphates such as IP_3_ based on scaffolds substituted with phosphate-recognition moieties such as guanidinium, alkyl ammonium or imidazolium groups as well as zinc(II) complexes. While these receptors have shown high affinity and, in some cases, good selectivity for specific IPs (mainly IP_3_), their binding to PIPs (i.e. the key lipid-derivatives of biological relevance) has not been reported. To address the lack of small-molecule receptors for PIPs, we previously developed a small-molecule (PHDM) able to bind PI(4,5)P_2_ with high selectivity both in vitro and in cellulo^17^. To the best of our knowledge, this is the only example of a small-molecule receptor that binds selectively to a PIP (rather than an IP) and is able to compete with protein domains in a cellular environment. Inspired by these previously reported scaffolds for IP_3_ as well as the recognition units in our earlier work with the linear PHDM receptor, herein we report a new tripodal molecule (**11**—see Fig. 2) able to bind to PI(3)P and PI(3,4)P_2_ selectively over several other mono-, bi- and tri-phosphorylated PIPs in a cell free environment. This receptor features two phenyl-thioureas, which are well-established recognition groups for oxoanions such as phosphates^18–20^. An ethyl-morpholino group was added in the *para* position of each of these phenyl-thioureas to increase the water solubility of receptor **11**. The third ‘arm’ of the tripodal receptor is an amino-boronic acid which can potentially display three types of interactions: (1) form reversible covalent bonds with diols present in the corresponding PIPs^21–23^; (2) display hydrogen bonding interactions with the phosphate groups^24, 25^; (3) form a covalent bond with an oxygen from one of the phosphate groups (with the concomitant change of geometry of the boronic centre to a tetrahedral centre)^26^. The affinity and selectivity of **11** against several PIPs was studied experimentally and rationalised via DFT calculations.

## Results and discussion

### Synthesis of receptor

In order to synthesise receptor **11**, it was first necessary to prepare starting materials **3**, **5** and **9** (Fig. 2). Compound **3** was synthesised following a previously reported synthetic procedure while compounds **5** and **9** (which had been previously reported) were prepared via a different procedure which gave good yields. These three compounds were characterised by ^1^H and ^13^C NMR spectroscopy, and by mass spectrometry (see ESI); all the data was consistent with that previously reported for each of the three compounds.

With the key intermediate **3** in hand, the formation of the thioureas on the two free amine arms was investigated. **3** was added to a solution of 1,1′-thiocarbonyldiimidazole (TCDI) and the reaction monitored by TLC over time. Once all of **3** had been consumed, amine **5** was added to the reaction mixture to yield the di-thiourea compound **6** in good yields (ranging between 77 and 86%). This compound was subsequently treated with TFA to remove the BOC protecting group to yield compound **7** (see Fig. 2). ^1^H NMR spectroscopy confirmed the formation of this new compound: the benzylic CH_2_ groups attached to the central scaffold and adjacent to the thiourea shifted downfield from 3.8 (in **3**) to 4.7 ppm (in **7**). The ^1^H NMR spectrum also showed the loss of the singlet at 1.4 ppm corresponding to the CH_3_ groups of the Boc-protecting group. The integration between the resonances in the aromatic and aliphatic protons was consistent with the proposed formulation of **7**. Furthermore, a resonance at 182 ppm in the ^13^C NMR spectrum was assigned to the C=S group confirming the formation of the thioureas, while the disappearance of the carbonyl peak at 158 ppm and the *t*-butyl CH_3_ carbon signals at 28 ppm confirmed deprotection of the amine. The sample was also analysed by ESI(+)-MS which confirmed that this intermediate product had formed (m/z = 778.4 a.m.u.).

Synthesis of **11** was achieved by alkylation of **7** with **9**, followed by deprotection of the boronate ester. ^1^H NMR spectroscopy of **11** confirmed the loss of the boronate ester singlet at 1.4 ppm as did the ^13^C NMR spectrum, which no longer showed the signal at 20 ppm. Furthermore, the ^1^H NMR spectrum showed a broad singlet at 9.2 ppm corresponding to the boronic acid OH-groups. All the expected aromatic and aliphatic resonances were present with the right integration for the proposed formulation, which was further confirmed by COSY and HSQC (see Supplementary Information). The ESI(+)-MS was also consistent with the proposed formulation of **11**, with a molecular peak at 962.5 a.m.u. which corresponds to [MH]^+^.

### Phosphatase assay to determine affinity of 11 for PIPs

Having successfully synthesised the new tripodal receptor **11**, we then investigated its binding affinity towards different PIPs in a cell-free environment. Most competition assays would either employ specific inositol lipid binding proteins or phosphatases, which have the caveat of a narrow substrate selectivity warranting the use of several different proteins/phosphatases to analyse all inositol lipids^17^, making it difficult to compare the data between the tested inositol lipids. To avoid this issue, we developed a new method that uses the same competing enzyme for all tested lipids allowing for direct comparison of the affinity of **11** to the seven PIPs. Alkaline phosphatase, which is used widely to dephosphorylate proteins and nucleic acids, has been utilised to hydrolyse glycerophosphoinositol phosphates^27^. We show here that this enzyme hydrolyses all phosphorylated inositol lipids in vitro (see ESI), making it an ideal tool to monitor inositol lipid binding in competition assays. Employing preincubated lipid/receptor **11** complexes lowers the freely available inositol lipid concentration for the alkaline phosphatase. The receptor’s affinity for the tested PIPs inversely correlates with the phosphatase activity since it will behave like a competitive inhibitor to the enzyme. This approach has the added benefit—as compared to other more traditional methods to assess host–guest interactions—that the receptor’s affinity/selectivity is tested under more challenging conditions, namely in the presence of an enzyme that competes with receptor **11**.

**Figure 3:**
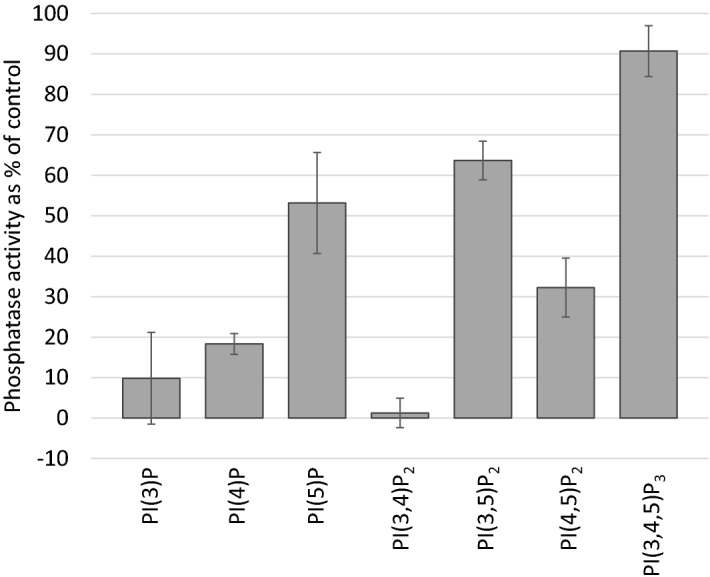
Binding specificity of receptor **11**. The affinity of **11** towards all seven naturally occurring inositol phospholipids was tested with a new alkaline phosphatase assay. The lipids were presented at a 40 µM concentration and incubated with alkaline phosphatase at a concentration adjusted for the different lipids. Receptor **11** was used at 62.5 µM. The absorbance is plotted as a percentage of control where no receptor is present (no receptor set at 100%). Data represented are the mean of two independent experiments performed in triplicates. Error bars represent ± standard deviation of two independent repeats carried out in triplicates (n = 6).

Figure 3 shows the activity of alkaline phosphatase towards each lipid substrate (40 µM) in the presence of excess amounts of tripodal receptor **11** (62.5 µM) in a cell-free environment. Mono- and bis-phosphorylated inositol lipids had much lower phosphatase activity than the tri-phosphorylated one, implying that receptor **11** binds reversibly to this group of lipids. Within each inositol lipid group a clear selectivity towards the 3- and 4-phosphorylation is detectable. The receptor shows the lowest affinity towards inositol lipids containing 5-phosphates (PI(5)P, PI(3,5)P_2_ and PI(4,5)P_2_). Interestingly, **11** shows the strongest binding affinity towards PI(3,4)P_2_, which results in almost complete inhibition of the enzyme’s activity. However, adding another 5-phosphate to this lipid (PI(3,4,5)P_3_) completely abolished receptor **11** binding, supporting the notion that the 5-phosphate position is a strong discriminator in the selectivity of the receptor.

**Figure 4:**
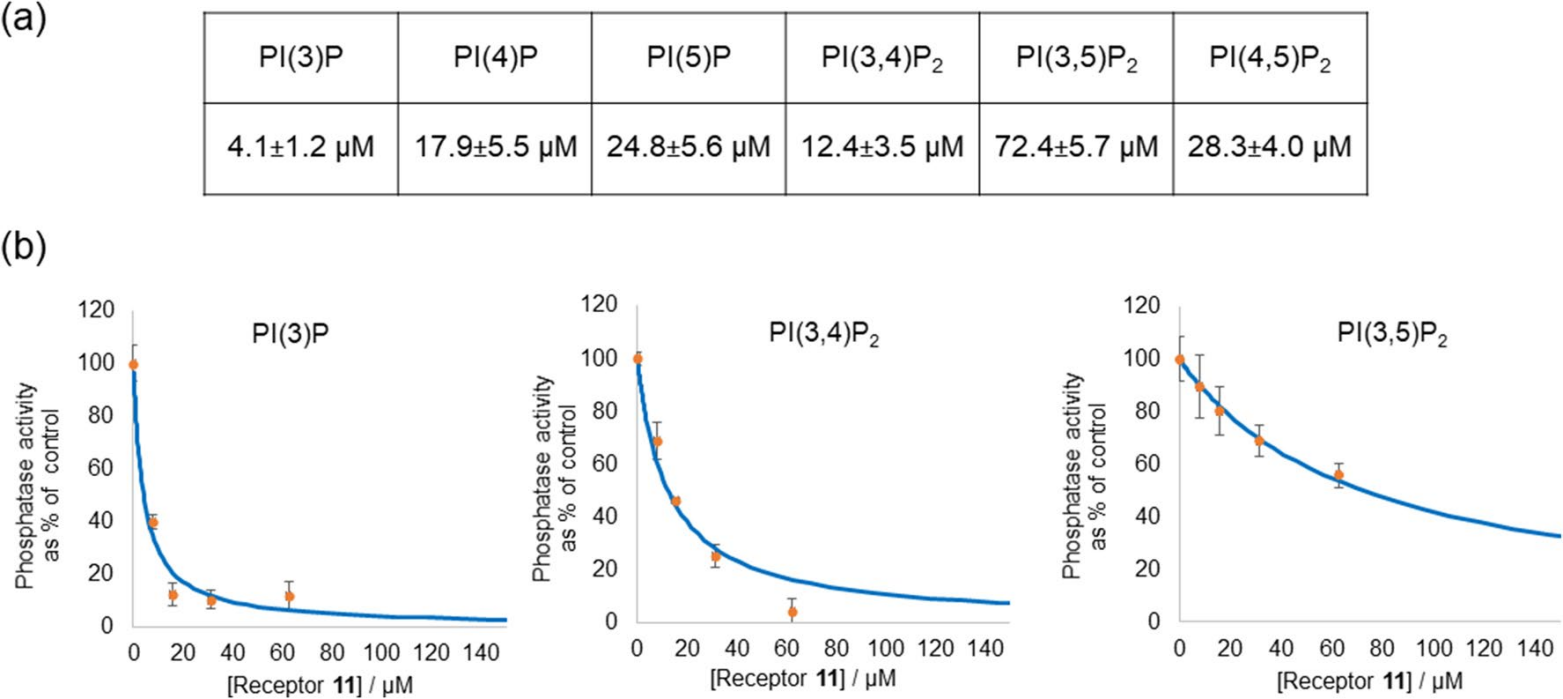
(**a**) IC_50_ values (µM) for the dephosphorylation of six different PIPs upon addition on increasing amounts of receptor **11**; the values shown are the average of three independent measurements with the corresponding error; (**b**) Representative examples of IC_50_ curves for the dephosphorylation of PI(3)P, PI(3,4)P_2_ and PI(3,5)P_2_ by alkaline phosphatase upon addition of increasing amounts of **11**.

Having established that the activity of receptor **11** is dependent on the inositol phosphate position, we determined the apparent IC_50_ values of **11** with each of the mono- and bi-phosphorylated PIPs (representative examples of IC_50_ curves are shown in Fig. 4 together with all values in the corresponding table; all other plots are shown in the Supplementary Information).

The results indicate that under the conditions of this cell-free test receptor **11** has the highest affinity for PI(3)P, followed by PI(3,4)P_2_, PI(4)P, PI(5)P, PI(4,5)P_2_ and PI(3,5)P_2_. There seems to be a clear preference for the 3-phosphate position, but also a clear avoidance of the 5-position. There also seems to be a preference for biphosphorylated PIPs where the phosphate are vicinal—cf. PI(4,5)P_2_ versus PI(3,5)P_2_. Given that both phosphate positions are equatorial and equidistant from the phosphodiester in the inositol lipid, this apparent regioselectivity of the receptor is intriguing and prompted us to investigate this further by computer modelling.

### Structure and energetics of receptor 11 complexed with PIP’s

**Figure 5:**
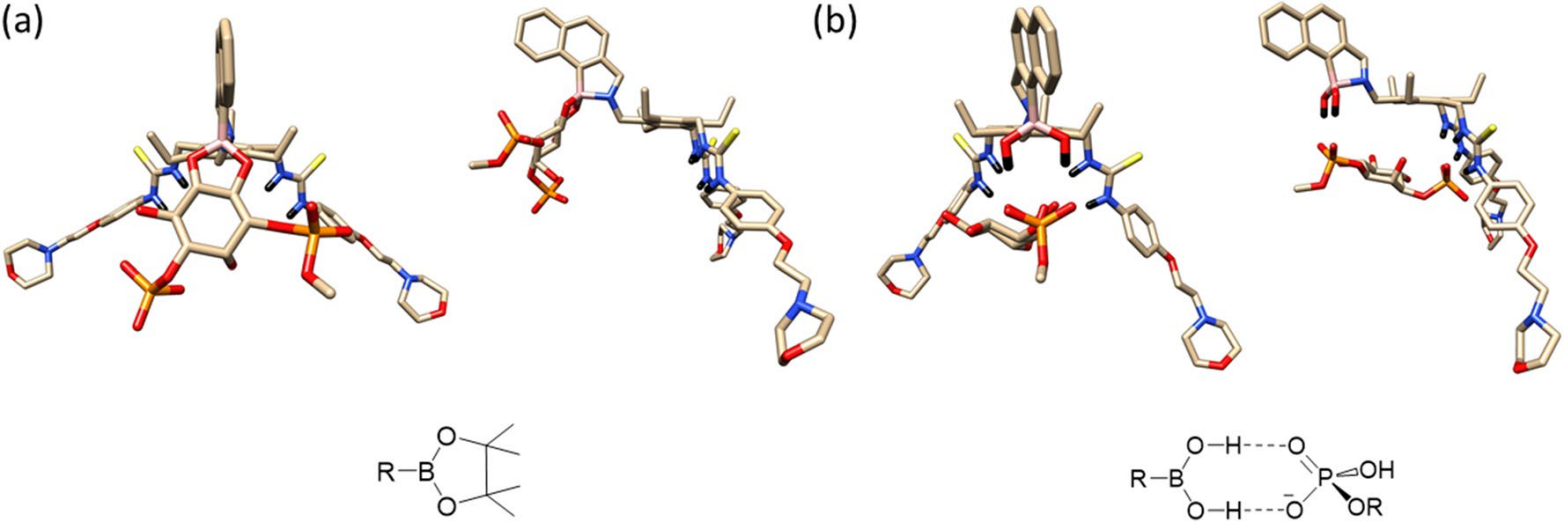
Fully optimised structures of the mono-phosphorylated PI(3)P with receptor **11** bound (**a**) covalently (i.e. via B–O bonds); and (**b**) displaying only non-covalent interactions.

To rationalise the selectivity profile of receptor **11** and to determine the structure and energetics of the host–guest interactions, DFT molecular modelling investigations were performed. As discussed in the introduction, boronic acids can either interact via reversible covalent bonds with the PIPs' diols or via non-covalent hydrogen bonding interactions with the phosphate groups (see Fig. 5). Therefore, we first explored these two possible binding modes between the B(HO)_2_ group of receptor **11** and PIPs using a model of PI(3)P. We note that to reduce computational time, all calculations presented in this section, were performed with simplified versions of PIPs in which the long lipid chain on the phosphoester (see Fig. 1) was substituted by a methyl group.

The optimised structures exploring the two binding modes mentioned above (Fig. 5) showed some striking differences. It is clear that for the covalently bonded case (a) there is no possibility of the PI(3)P interacting with the thiourea groups of the receptor whilst for the non-covalently bonded case (b) there is a clear interaction of the PI(3)P phosphate with the thioureas. Geometries were located (identified as local minima) for all the mono-phosphorylated PIP’s for the non-covalent and covalently bound systems. For the three bi-phosphorylated PIPs it was possible to locate structures for the non-bonded systems and for two of the bonded systems. However, it was not possible to locate an optimised structure for PI(3,5)P_2_. For the non-bonded PI(4,5)P_2_ and bonded PI(3,4)P_2_ structures, these were identified as very weak transition states with all other structures being identified as true minima. The difference between the two possible binding modes is confirmed when considering the relative energetics of the two systems (see Table 1).

**Table 1:** Interaction energies for the mono-phosphate PIP’s. The asterix next to a system indicates that it was characterised as a transition state, whilst those without an asterisk where confirmed as local minima by vibrational frequency analysis.

	ΔH kJ/Mol	ΔG kJ/Mol
**Non-bonded system**		
PI(3)P	− 127.38	− 35.42
PI(4)P	− 119.20	− 34.08
PI(5)P	− 102.40	− 18.82
PI(3,4)P_2_	− 118.73	− 9.48
PI(4,5)P_2_*	− 115.16	− 18.34
PI(3,5)P_2_	− 107.64	− 20.93
**Bonded system**		
PI(3)P	+ 28.08	− 9.51
PI(4)P	+ 40.61	+ 3.28
PI(5)P	+ 16.67	− 12.04
PI(3,4)P_2_*	+ 72.30	+ 43.61
PI(4,5)P_2_	+ 95.05	+ 63.72
PI(3,5)P_2_	N/A	N/A

These calculations established that the binding mode between receptor **11** and the PIPs is unlikely to involve reversible B–O covalent bonds and be dominated by non-covalent interactions. The complete computational details and PDB’s of the optimised structures are given in the supplementary material.

**Figure 6:**
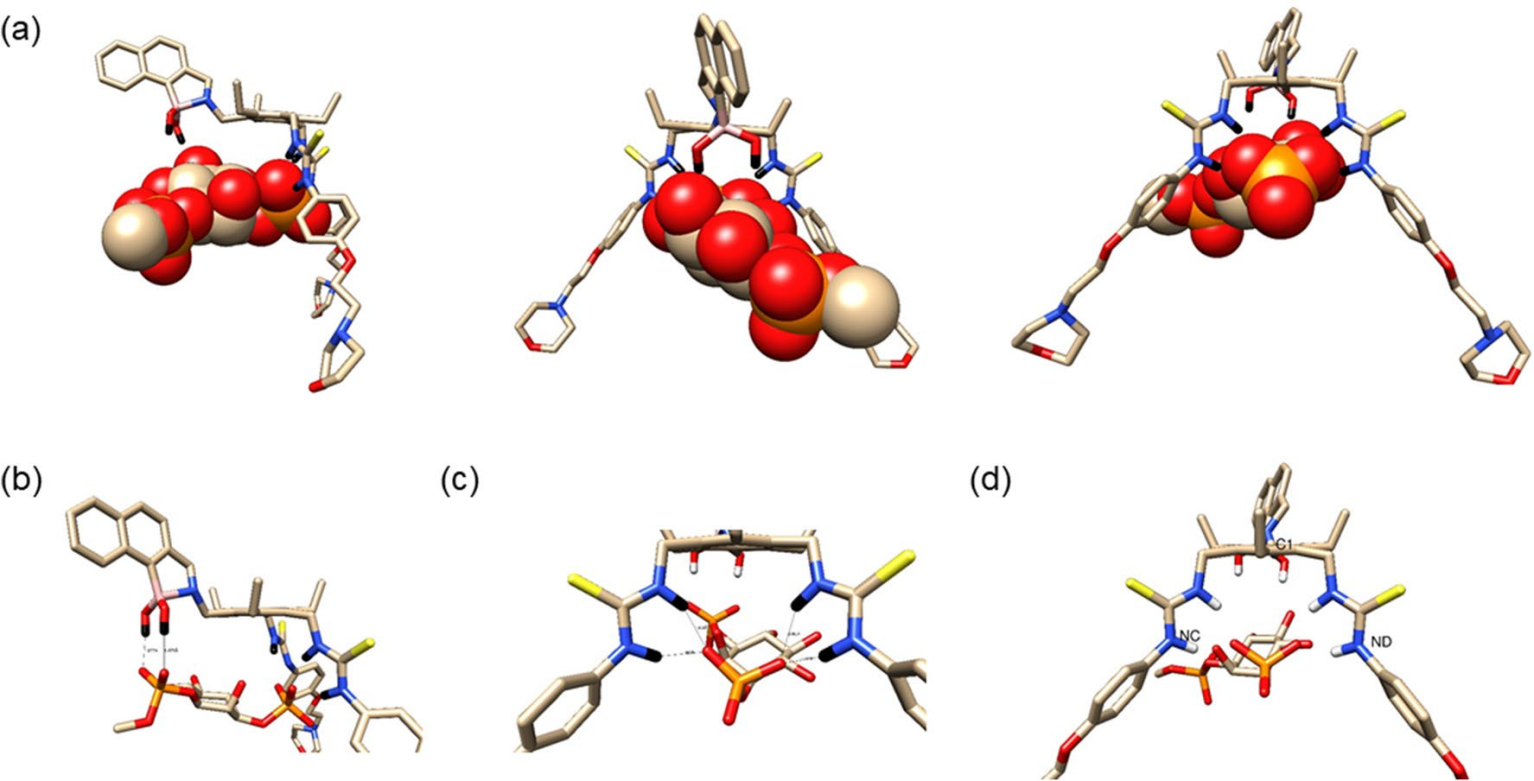
(**a**) Fully optimised structures of the mono-phosphorylated PI(3)P with receptor **11** side, head-on and from behind views (left to right respectively). Hydrogen-bonding definitions for: (**b**) lipid phosphate end of PI(3)P and B–N end of receptor; (**c**) phosphate of PI(3)P ring and thiourea groups of the receptor. (**d**) Definition of the bite angle atoms of PI(3)P, NC-C1-ND.

For the non-covalent mono-phosphorylated PIP’s, Fig. 6 illustrates the results for PI(3)P as a representative example (the structure for PI(4)P and PI(5)P can be found in the ESI).

The results reveal that the receptor can form stable complexes with all three mono-phosphorylated PIPs and the complexes formed have clear structural similarities (see Fig. 6 and ESI). In Table S1 we highlight these similarities with respect to key H-bond distances and the “bite” angle of the receptor. From this data there is little to differentiate the distance of the PIP’s to the receptor, however, there is a clear closing of the receptor “bite” angle when a PIP is complexed with it; the “bite” angle for the receptor on its own is 99.6°. The phosphoester group of all three monophosphorylated PIP’s form two hydrogen bonds with the boronic acid's hydrogens. For the phosphate group on the inositol ring all three PIP’s hydrogen-bond to the receptor in the same way, i.e. there are two hydrogen bonds formed from the O3 atom of the phosphate to the HA and HC hydrogens of the receptor and this is mirrored by the two hydrogen bonds formed from the phosphate O5 atom to the HB and HD hydrogens.

Irrespective of which interaction energy metric is used, ΔH or ΔG, from the data shown in Table 1, it is clear that the most stable complex is that formed between **11** and PI(3)P, with PI(4)P being the second strongest binder to **11**, and PI(5)P the weakest of the three in line with the experimental findings.

**Figure 7:**
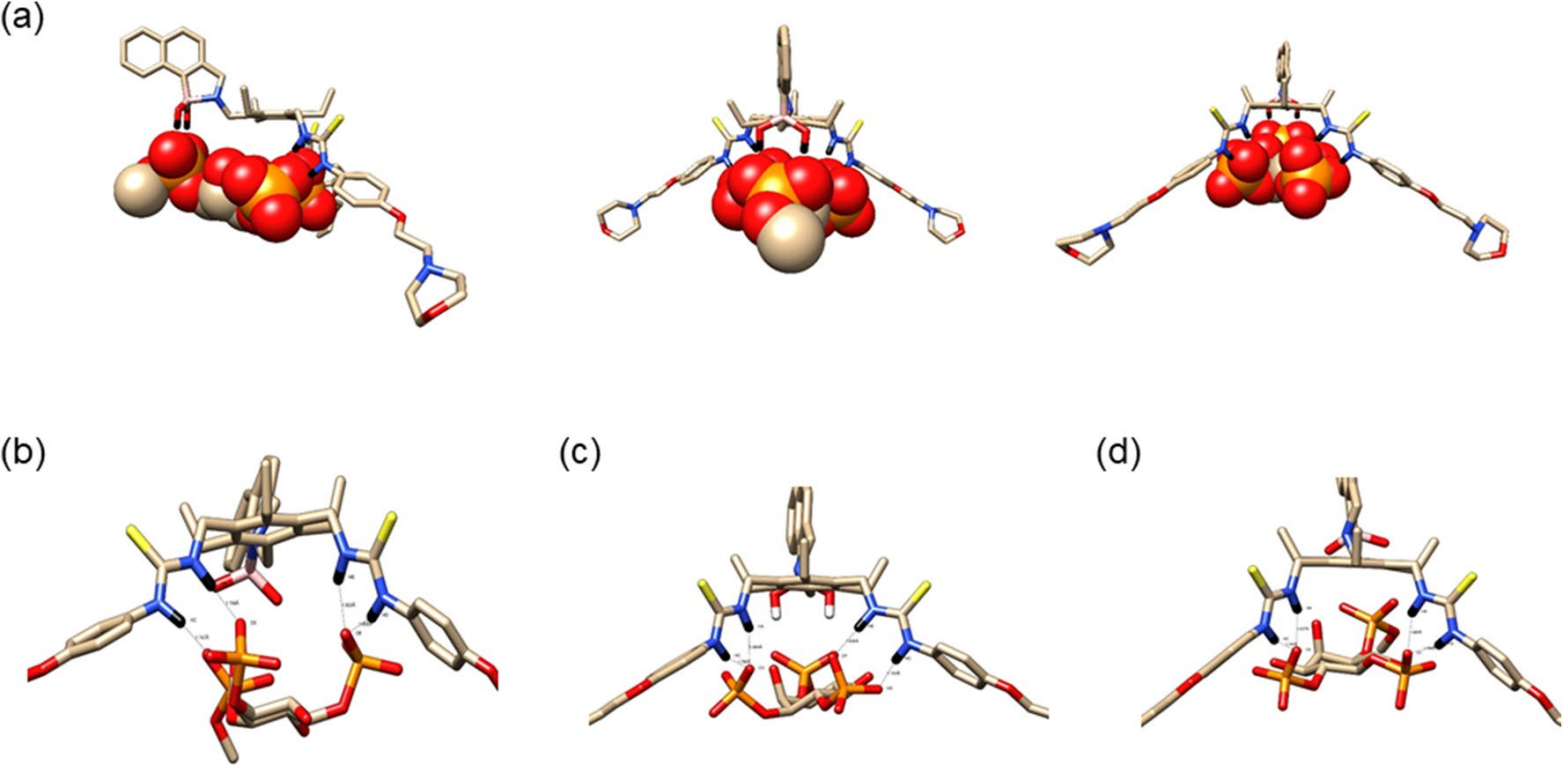
(**a**) Complexes between **11** and PI(3,4)P_2_, are presented from a side, top and back perspective when viewed left to right. Hydrogen-bonding definitions for receptor **11** with (**b**) PI(3,4)P_2_; (**c**) PI(3,5)P_2_ and (**d**) PI(4,5)P_2_.

For the bi-phosphorylated complexes we observed similar binding motifs as seen for the mono-phosphorylated complexes. Figure 7a reports three views (side, top and back) for the fully optimized structures of the complex between **11** and PI(3,4)P_2_ (and for PI(4,5)P_2_ and PI(3,5)P_2_ the figures are shown in the ESI). PDB’s of the complexes supplied in the supplementary material should facilitate a more accessible method to assess the geometries.

Geometric analysis of the complexes for the bi-phosphorylated PIP complexes is more challenging than for the mono-phosphorylated ones, due to the addition of a second phosphate on the PIP inositol ring. Therefore, we illustrate the differences in the final structures from the back perspective in Fig. 7b–d.

In Table S1 we report the same structural data as for the mono-phosphorylated PIP’s, however, these data are best examined by referencing the PDB’s supplied in the supplementary information. One consistent observation is that the “bite” angle for all three complexes has increased going from mono- to bi-phosphorylated PIPs. For the complex of **11** with PI(4,5)P_2_ the hydrogen-bonding pattern for the inositol phosphates is similar to that of the mono-phosphates, the HA and HC of the receptor both hydrogen bond to a single oxygen, O5, of the phosphate at position 4 of the ring and the HB and HD with the O7 of the phosphate at position 5. For PI(3,4)P_2_ and PI(3,5)P_2_ the hydrogen boding motifs of the inositol phosphates are mirrors of each other, for PI(3,4)P_2_ HA and HC both hydrogen bond to O3, whilst for PI(3,5)P_2_ HB and HD both hydrogen bond to O8; in PI(3,4)P_2_ HB and HD hydrogen bond individually with O7 and O6 of the inositol ring respectively whilst for PI(3,5)P_2_ HA and HC hydrogen bond individually with O5 and O3 respectively.

As for the mono-phosphorylated PIP’s, we have also evaluated the relative energy ordering of the complexes with the bi-phosphorylated PIP’s. The ΔH interaction energies reveals the same order of binding PI(3,4)P_2_ > PI(4,5)P_2_ > PI(3,5)P_2_ as the experimental IC_50_ data, however, this order is reversed when the ΔG’s are evaluated, PI(3,5)P_2_ > PI(4,5)P_2_ > PI(3,4)P_2_. The origin of this difference is the entropy contribution, in the case of PI(3,4)P_2_ the PIP is closely bound to **11** therefore the complex entropy is smaller compared to that for PI(4,5)P_2_ and PI(3,5)P_2_ which are less tightly bound and therefore have higher entropies.

## Conclusions

We have successfully prepared a new tripodal molecular receptor (**11**) via a multi-step synthesis. This receptor can be prepared in good yields and displays good solubility in aqueous buffer. In order to evaluate the affinity of the new receptor for PIPs in a functional manner (in a cell-free environment), we have developed a novel alkaline phosphatase assay in which the activity of the enzyme can be directly correlated to the affinity of the receptor for the headgroup of the PIP substrate. The receptor displays good selectivity towards PI(3)P and PI(3,4)P_2_ over five other PIPs—with particularly low binding observed for PI(5)P and PI(3,5)P_2_, and no binding observed for PI(3,4,5)P_3_. This new receptor is, to the best of our knowledge, the first example of a synthetic compound that can selectively recognise PI(3,4)P_2_ and PI(3)P. Although our initial studies have been carried out in a cell-free environment, compound **11** provides a good scaffold for the future development of other PIP receptors which might display interesting cellular properties.

To rationalise the experimental data, DFT modelling studies were performed, which indicated that all three mono- and three bi-phosphorylated PIPs are capable of forming complexes with receptor **11**. Interestingly, these modelling studies have clearly shown that the preferred binding mode between the receptor and the PIPs does not involve the formation of reversible B–O bonds. Instead, the receptor displays a number of hydrogen bonding interactions with the PIPs which drives the observed selectivity. The modelling correctly predicts that PI(3)P forms the strongest interaction closely followed by PI(4)P, irrespective of whether ΔH or ΔG is the metric. Our DFT calculations indicated that the PI(5)P and the PI(3,5)P_2_ complexes are the weakest formed when considering their ΔH of interaction, which is consistent with the experimental data. The correct ordering of the complexation energy, ΔH, of **11** with PI(3,4)P_2_ and PI(4,5)P_2_ is observed with PI(3,4)P_2_ being the stronger binder of the two. The DFT studies have allowed us to establish a proposed binding mode between the PIPs and **11** as well as providing an overall explanation for the differences in complexation ability.

## Methods

### General information

^1^H NMR, ^13^C NMR and ^31^P NMR spectra were recorded on either a Bruker Avance 400 MHz Ultrashield NMR spectrometer or a Bruker Avance 500 MHz NMR spectrometer (for complete assignment of ^1^H and ^13^C NMR spectra see ESI). Electrospray ionisation mass spectra were obtained on a Bruker Daltonics Esquire 3000 spectrometer. Compounds **1**^28^, **2**^29^ and **3**^30^ were prepared following previously reported procedures (see ESI). While compounds **5**, **8** and **9** have been previously reported^31^, they were prepared by different routes as detailed in the ESI.

### Synthesis of compound 6

Diamine **3** (332 mg, 0.95 mmol) in anhydrous MeCN (13 mL) was added dropwise to a stirring solution of 1,1′-thiocarbonyldiimidazole (342 mg, 1.92 mmol) in anhydrous MeCN (13 mL) cooled in an ice-water bath. The reaction mixture was stirred for 30 min in an ice-bath under nitrogen before amine **5** (427 mg, 1.92 mmol) in anhydrous MeCN (10.0 mL) was added dropwise at a rapid rate. The reaction mixture was heated at reflux under nitrogen overnight. The reaction mixture was concentrated in vacuo and the residue was partitioned between CHCl_3_ (50 mL) and H_2_O (50 mL). The layers were separated and the aqueous layer back-extracted with CHCl_3_ (2 × 40 mL). The combined organic layers were washed with water (3 × 80 mL). The resulting aqueous layer was further back-extracted with CHCl_3_ (5 × 50.0 mL). The resulting combined organic layers were dried using Na_2_SO_4_ and following filtration under gravity concentrated in vacuo. Flash column chromatography eluting with CHCl_3_/MeOH (9.8:0.2) followed by CHCl_3_/MeOH (9.5:0.5) gave thiourea **6** as a pale orange solid foam (717 mg, 86%). TLC (CHCl_3_/MeOH (9:6:0.4): R_*f*_ = 0.18; ^1^H-NMR (400 MHz, CDCl_3_) δ 1.07 p.p.m. (3H, br t, *J* 7.2), 1.13 (6H, br t, *J* 7.4), 1.43 (9H, br s), 2.61–2.64 (12H), 2.84 (6H, br s), 3.74–3.75 (8H, J), 4.13 (4H, m), 4.28 (2H, br s), 4.75 (4H, br s), 6.87 (4H, d, *J* 8.7), 7.09 (4H, d, *J* 8.7); ^13^C-NMR (100 MHz, MeOD) δ 16.8, 16.9, 24.0, 28.8, 39.7, 44.4, 55.1, 58.7, 66.6, 67.6, 80.3, 116.0, 128.0, 132.8, 133.9, 145.5, 145.8, 158.0, 158.4, 182.4; m/z (electrospray) 878.4654 (MH^+^, 40%), 439.7334 (MH_2_^2+^, 100%) Found: MH^+^, 878.4654. C_46_H_68_N_7_O_6_S_2_ requires 878.4673; Δ = −2.2 ppm.

### Synthesis of compound 7

To a stirred solution of Boc-protected amine **6** (694 mg, 0.79 mmol) in anhydrous CH_2_Cl_2_ (18.00 mL) was added TFA (4.42 mL, 57.7 mmol). The reaction mixture was stirred at room temperature overnight. The reaction mixture was concentrated in vacuo. The residue was partitioned between CHCl_3_ (20 mL) and 2 M NaOH (20 mL). The layers were separated and the organic layer further washed with 2 M NaOH (20 mL) and H_2_O (20 mL). The aqueous layer was back-extracted with CHCl_3_ (3 × 40 mL) followed by a 3% MeOH in CHCl_3_ solution (3 × 40 mL). The combined organic layers were dried using Na_2_SO_4_ and filtered under gravity. Flash column chromatography eluting with CHCl_3_/MeOH (9.5:0.5), then CHCl_3_/MeOH/7N NH_3_ in MeOH (9.45:0.5:0.05) gave amine **7** as a white solid foam (497 mg, 81%). TLC (CHCl_3_/7N NH_3_ in MeOH (9:95:0.05)): R_*f*_ = 0.16; ^1^H-NMR (400 MHz, MeOD) δ 1.18 (9H, t, *J* 7.1), 2.57 (8H, m), 2.76 (10H, m), 3.69 (8H, m), 3.86 (2H, s), 4.11 (4H, t, *J* 5.5), 4.74 (4H, br s), 6.91 (4H, d, *J* 8.8), 7.22 (4H, d, *J* 8.8); ^13^C-NMR (100 MHz, MeOD) δ 16.9, 23.9, 39.4, 44.5, 55.1, 58.7, 66.7, 67.6, 116.1, 127.0, 132.4, 132.8, 137.7, 144.8, 145.4, 158.4, 182.4; m/z (electrospray) 778.4164 (MH^+^, 100%), 389.7068.2176 MH_2_^2+^, 40%) Found: MH^+^, 778.4164. C_41_H_60_N_7_O_4_S_2_ requires 778.4148; Δ = 2.1 ppm.

### Synthesis of compound 10

A solution of amine **7** (474 mg, 0.61 mmol) and K_2_CO_3_ (51.0 mg, 0.37 mmol) in anhydrous DMF (6 mL) was stirred under nitrogen for 30 min before a solution of bromine **9** (106 mg, 0.31 mmol) in anhydrous DMF (2.50 mL) was added dropwise over 25 min. The reaction mixture was stirred under nitrogen at room temperature for 6 h. The reaction mixture was concentrated in vacuo and the residue was partitioned between CHCl_3_ (15 mL) and brine (15.0 mL). The layers were separated and the aqueous layer back-extracted with CHCl_3_ (2 × 10 mL). The organic layers further were washed with brine (3 × 30.0 mL). The resulting aqueous layer was further back-extracted with CHCl_3_ (3 × 40.0 mL) followed by a 3% MeOH in CHCl_3_ solution (3 × 40 mL). The resulting combined organic layers were dried using Na_2_SO_4_ and following filtration under gravity concentrated in vacuo. Flash column chromatography eluting with CHCl_3_/MeOH (9.85:0.15), then CHCl_3_/MeOH (9.5:0.5) gave boronate ester **10** as an off-white foam solid (149 mg, 48%). TLC (CHCl_3_/MeOH (9:98:0.02)): R_*f*_ = 0.29; ^1^H-NMR (400 MHz, MeOD) δ 0.90 (6H, br t, *J* 7.2), 1.22 (3H, m), 1.46 (12H, s), 2.56 (12H, m), 2.78 (6H, m), 3.68 (8H, br t, *J* 4.6), 3.78 (2H, br s), 3.97 (2H, br s), 4.12 ( 4H, br t, *J* 5.4), 4.80 (4H, br s), 6.95 (4H, d, *J* 8.8), 7.13 (1H, br d, *J* 8.0), 7.24 (4H, br d, *J* 8.8), 7.44 (1H, br t, *J* 6.8), 7.50 (1H, br t, *J* 6.8), 7.81 (1H, d, *J* 8.0), 7.86 (1H, d, *J* 8.0), 8.6 (1H, d, *J* 8.1); ^13^C-NMR (100 MHz, MeOD) δ 16.5, 16.8, 24.2, 26.6, 43.8, 44.4, 49.0, 55.1, 58.7, 66.7, 67.6, 81.8, 116.1, 123.0, 126.2, 126.4, 128.0, 128.6, 129.6, 130.1, 130.4, 132.5, 133.3, 135.0, 136.8, 140.0, 146.7, 147.1, 158.5, 182.4; m/z (electrospray) 1044.5657 (MH^+^, 100%), 522.7809 MH_2_^2+^, 70%) Found: MH^+^, 1044.5657. C_58_H_79_N_7_O_6_S_2_11B requires 1044.5626; Δ = 3.0 ppm.

### Synthesis of compound 11

Boronate ester **10** (120 mg, 0.12 mmol) was stirred in a 70% TFA: H_2_O solution (5.80 mL) at 68 °C for 24 h. The solvent was removed in vacuo. The residue was taken up in CHCl_3_ (10 mL) and washed with 2 M NaOH (2 × 10 mL). The aqueous layer was further extracted with CHCl_3_ (5 × 7 mL). The combined organic layers were concentrated in vacuo. The resulting precipitate was washed several times with methanol and isolated the centrifuge. This process gave boronic acid **11** as an off-white powder (65 mg, 59%). ^1^H-NMR (400 MHz, DMSO) δ 1.13 (6H, br t, *J* 7.2), 1.20 (3H, br t, *J* 7.3), 2.49 (8H, m), 2.69 (4H, t, *J* 5.8), 2.78 (2H, br d, *J* 7.3), 2.97 (6H, m), 3.21 (1H, s), 2.59 (8H, m), 3.92 (2H, br s), 4.07 (4H, t, *J* 5.8), 4.72–4.74 (6H), 6.86 (4H, d, *J* 9.0), 7.06 (2H, m), 7.34 (4H, d, *J* 9.0), 7.39 (1H, br d, 8.3), 7.44 (1H, m), 7.51 (1H, m), 7.80 (1H, m), 7.87 (1H, m), 8.55 (1H, d, *J* 8.2), 9.04 (2H, br s); ^13^C-NMR (100 MHz, DMSO) δ 15.5, 15.6, 21.7, 22.1, 41.2, 42.2, 51.3, 53.1, 56.6, 65.7, 114.2, 120.6, 124.2, 124.6, 125.1, 126.4, 127.4, 128.1, 131.4, 131.6, 132.2, 132.8, 134.0, 142.8, 143.6, 148.8, 155.2, 180.7; LCMS trace 100%; m/z (electrospray) 962.4866 (MH^+^, 100%), 944.4828 (MH^+^–OH, 35%) Found: MH^+^, 962.4866. C_52_H_69_N_7_O_6_S_2_11B requires 962.4844; Δ = 2.2 ppm.

### Experimental details for the alkaline phosphatase (AP) assay with lipid substrates

All seven PIPs were presented as mixed micelles with a final octyl-glucoside concentration of 0.2% (v/v). For the calibration curve lipid solutions of increasing concentration were incubated with AP (5 or 0.5 nM, see below) in a 96-well plate for 30 min at 37 °C in the presence of 100 mM TRIS, pH 6.8 and a total volume of 50 µL per well. The reaction was terminated by addition of an equal volume of phosphate detection reagent (50 µL) to quantify the amount of phosphate released as described before. The rate of the enzyme activity was determined as Δ absorbance of the reaction minus the enzyme control (enzyme was added after the phosphate detection reagent). The assay was optimised for each individual PIP to have comparable rates in the linear range by adjusting the enzyme concentration (see Figure S35). Subsequently, AP concentration of 5 nM was used for monophosphorylated lipids PI(3)P, PI(4)P and PI(5)P as well as PI(3,4)P_2_ and 0.5 nM for PI(4,5)P_2_, PI(3,5)P_2_ and PI(3,4,5)P_3_.

For testing the binding affinity of the receptors, the AP assay was modified into a 2-step procedure, with the first step being used to form lipid-receptor complexes and the second step being the application of the AP assay to determine the free lipid concentration. For the first step, receptors were preincubated with a constant concentration of PIP (40 µM) for 1 h in the presence of 100 mM TRIS, pH 6.8, 0.2% (v/v) OG and 2% (v/v) DMSO in a total volume of 50 µL. This was followed by the addition of AP (second step) in a 96-well plate for 30 min at 37 °C. The reaction was terminated by addition of and equal volume of phosphate detection reagent based on malachite green (50 µL) to quantify the amount of phosphate released. The turnover of the enzyme was determined as described above.

### Computational studies

Initial structures of the PIP’s and the receptor were constructed using the model building tools in Gaussview 6.1 (Version 6.1, Roy Dennington, Todd A. Keith, and John M. Millam, Semichem Inc., Shawnee Mission, KS, 2016). These structures were then fully geometry optimised at the B3LYP level of theory with the 6–31++g(d,p) basis set in water, using the default SCRF = PCM model, with Gaussian 16^32^; all standard default settings were used in the optimisation process. Models were then constructed of covalently and non-covalently bonded complexes of the receptor with the individual PIP’s, all resulting complexes were then full geometry optimised at the same level as the individual PIP’s and receptor. All structures reported, PIP’s, receptor and complexes, were identified as minima or transition states through the calculation of the vibrational spectra. All images rendered in the manuscript and in the ESI were performed with the UCSF-Chimera program^33^. PDB structures of the optimised systems, including the bonded complexes, are also provided.

## Supplementary information

Supplementary Information 1.

Supplementary Information 2.
