# Supplementary material for: Design, synthesis and evaluation of a tripodal receptor for phosphatidylinositol phosphates
Source: Sci Rep. 2020 Oct 28;10:18450. doi: 10.1038/s41598-020-75484-w (PMC7595110; doi:10.1038/s41598-020-75484-w)
Supplement: Supplementary file 2 — Supplementary Information 2. [file 41598_2020_75484_MOESM2_ESM.pdf]

Design, synthesis and evaluation of a tripodal receptor for phosphatidyl inositol phosphates

Katharina Reeh, Peter Summers, Ian Gould, Rudiger Woscholski and Ramon Vilar*

Department of Chemistry, Imperial College London, White City Campus, Wood Lane, London W12 OBZ, UK

Institute of Chemical Biology, Imperial College London, London SW7 2AZ, UK

Corresponding author: r.vilar@imperial.ac.uk

Supplementary Information

Synthesis of the tripodal receptor 11.

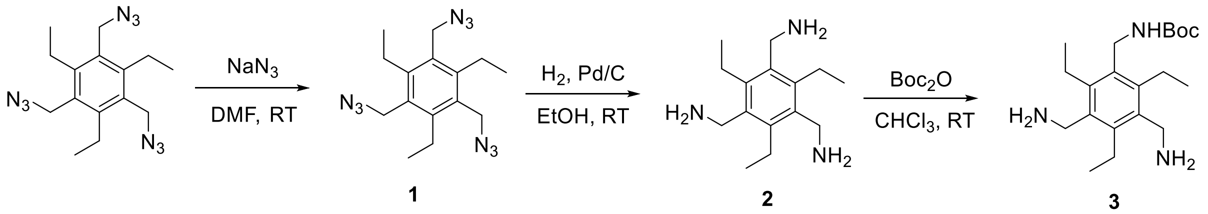

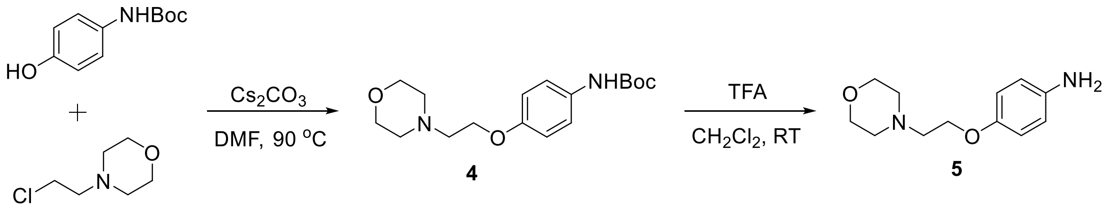

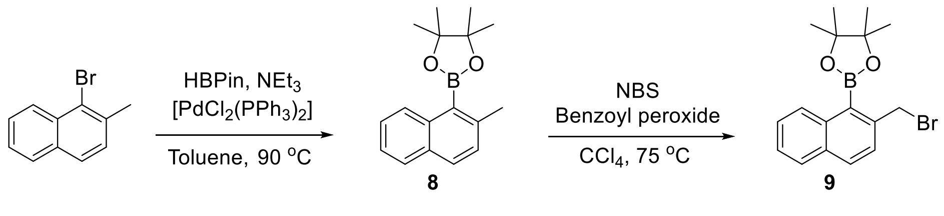

Synthesis of tert-butyl N-{4-[2-(morpholin-4- yl)ethoxy]phenyl}carbamate (4).

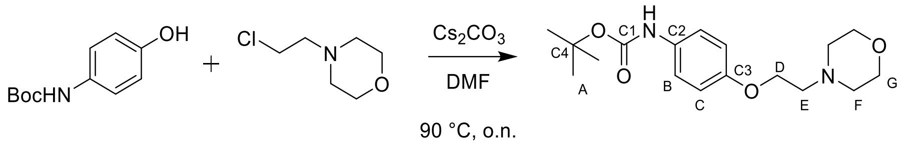

4

A suspension of tert-butyl N-(4-hydroxyphenyl)carbamate (250 mg, 1.20 mmol), 4-(2-chloroethyl)morpholine hydrochloride (245 mg, 1.31 mmol) and Cs_2CO_3 (856 mg, 2.63 mmol) in anhydrous DMF (15.0 mL) was heated at 90 °C for 18 hr under nitrogen. The reaction mixture was diluted with EtOAc (15.0 mL) and partitioned with water (20.0 mL). The layers were separated and the aqueous layer back-extracted with EtOAc (2 × 15.0 mL). The combined organic layers were washed with water (5 × 30.0 mL), dried using Na_2SO_4 and following filtration under gravity concentrated *in vacuo*. Flash column chromatography eluting with EtOAc/hexane (1:1) then EtOAc/hexane (4:1) gave ethoxy **4** as a white solid (296 mg, 79 %). TLC (EtOAc:hexane, 4:1 v/v): R_f = 0.20; ^{1}H-NMR (400 MHz, $CDCl_3$) δ 1.50 p.p.m. (9H, d, A), 2.57 (4H, br t, *J* 4.5, F), 2.87 (2H, t, *J* 5.7, E), 3.73 (4H, br t, *J* 4.5, G), 4.07 (2H, t, *J* 5.7, D), 6.34 (1H, br s, NH), 6.84 (2H, d, *J* 8.8, C), 7.25 (2H, br d, *J* 8.8, B); ^{13}C-NMR (100 MHz, $CDCl_3$) δ 28.5 p.p.m. (A), 54.2 (F), 57.8 (E), 66.2 (D), 67.0 (G), 80.4 (C4), 115.1 (C), 120.6 (B), 131.8 (C3), 153.4 (C2), 154.9 (C1); m/z (electrospray) 323.1964 (MH^+, 100%), Found: MH^+, 323.1964. $C_{17}H_{26}N_2O_4$ requires MH^+, 323.1971; Δ = -2.2ppm).

Spectra for tert-butyl N-{4-[2-(morpholin-4- yl)ethoxy]phenyl}carbamate (4).

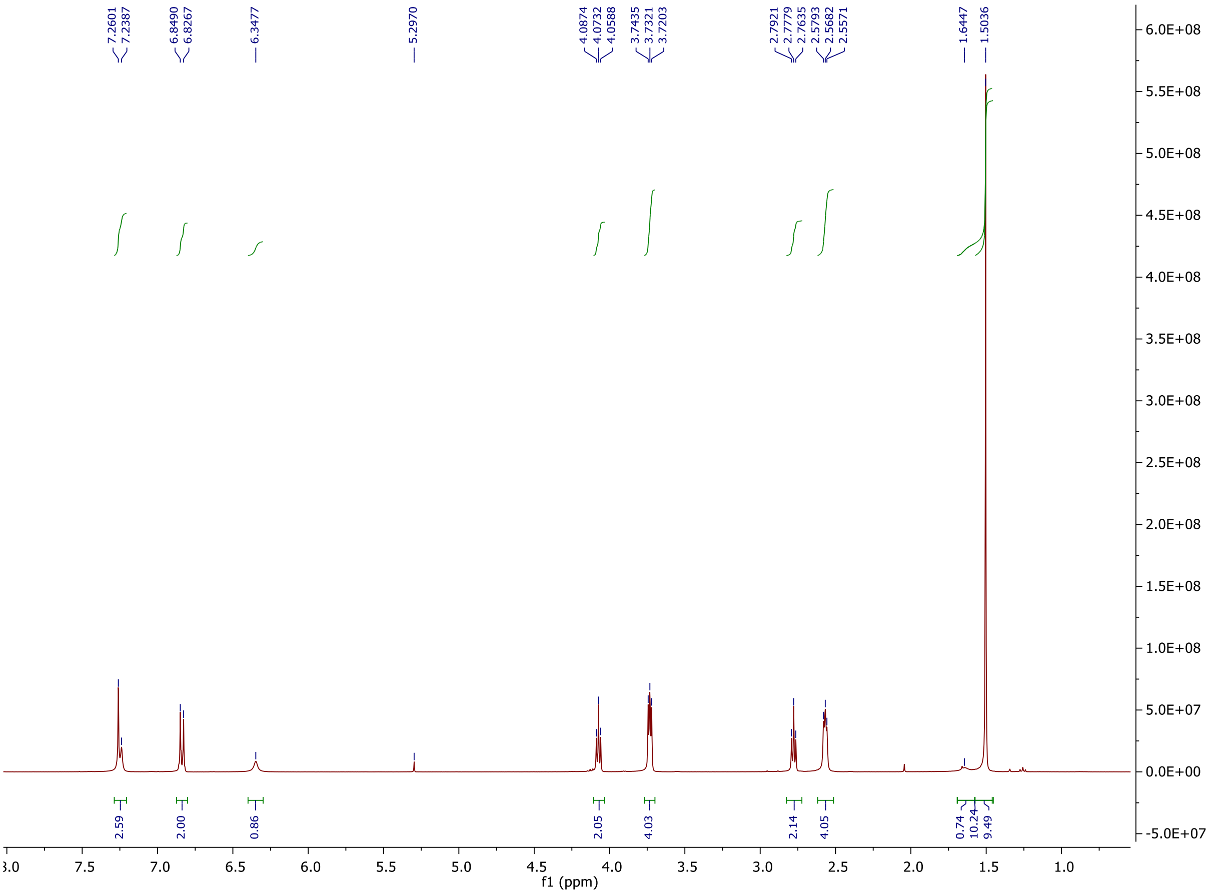

Figure S1. **^{1}H NMR spectrum of tert-butyl N-{4-[2-(morpholin-4- yl)ethoxy]phenyl}carbamate (4).**

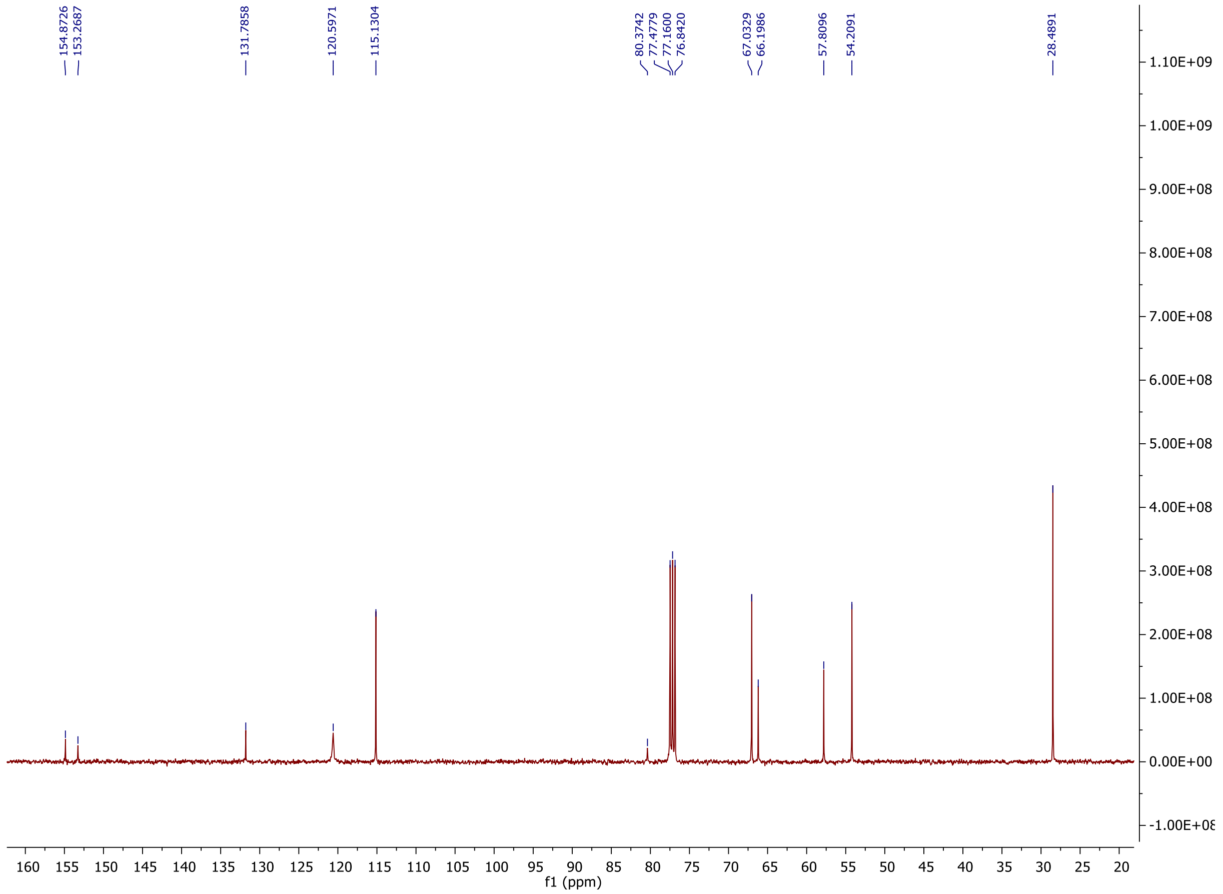

Figure S2. ^{13}C NMR spectrum of tert-butyl N-{4-[2-(morpholin-4- yl)ethoxy]phenyl}carbamate (4).

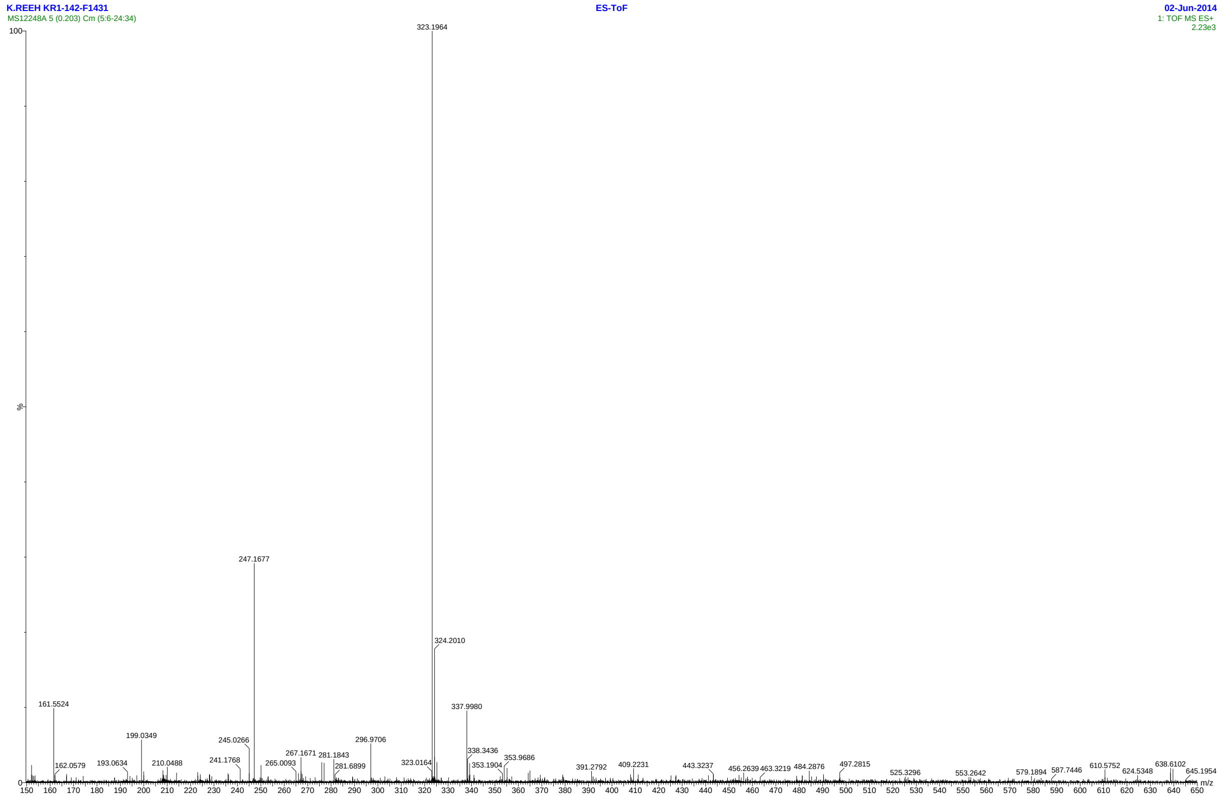

Figure S3. MS spectrum of tert-butyl N-{4-[2-(morpholin-4- yl)ethoxy]phenyl}carbamate (4).

Synthesis of 4-[2-(morpholin-4-yl)ethoxy]aniline (5).

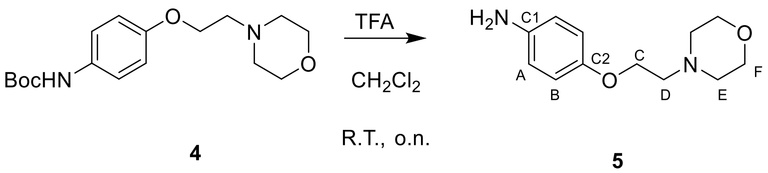

4 5

To a stirred solution of Boc-protected amine **4** (1.32 g, 4.09 mmol) in anhydrous CH_2Cl_2 (20.00 mL) under nitrogen was added TFA (3.56 mL, 46.5 mmol). The reaction mixture was stirred at room temperature under nitrogen overnight. The reaction mixture was concentrated *in vacuo*. The residue was partitioned between $CHCl_3$ (20.0 mL) and $NaHCO_3$ (20.0 mL). The layers were separated and the aqueous layer back-extracted with $CHCl_3$ (2 × 20.0 mL). The combined organic layers were washed with $NaHCO_3$ (2 × 30.0 mL) and water (3 × 30.0 mL). The resulting aqueous layer was back-extracted with $CHCl_3$ (6 × 25.0 mL). The combined organic layers were dried using Na_2SO_4 and filtered under gravity. Concentration of the filtrate gave amine **5** as a pale brown oil (874 mg, 94 %). TLC (EtOAc): R_f = 0.18; ^{1}H-NMR (400 MHz, $CDCl_3$) δ 2.56 p.p.m. (4H, br t, *J* 4.5, E), 2.76 (2H, t, *J* 5.8, D), 3.42 (2H, br s, NH_2), 3.73 (4H, br t, *J* 4.5, F), 4.03 (2H, t, *J* 5.8, C), 6.63 (2H, d, *J* 8.8, B), 6.75 (2H, br d, *J* 8.8, A); ^{13}C-NMR (100 MHz, $CDCl_3$) δ 54.2 p.p.m. (E), 57.9 (D), 66.5 (C), 67.0 (F), 115.9 (B), 116.5 (A), 140.3 (C1), 152.0 (C2); m/z (electrospray) 223.1438 (MH^+, 100%), Found: MH^+, 223.1438. $C_{12}H_{18}N_2O_2$ requires MH^+, 223.1447; Δ = -4.0ppm).

Spectra for 4-[2-(morpholin-4-yl)ethoxy]aniline (5).

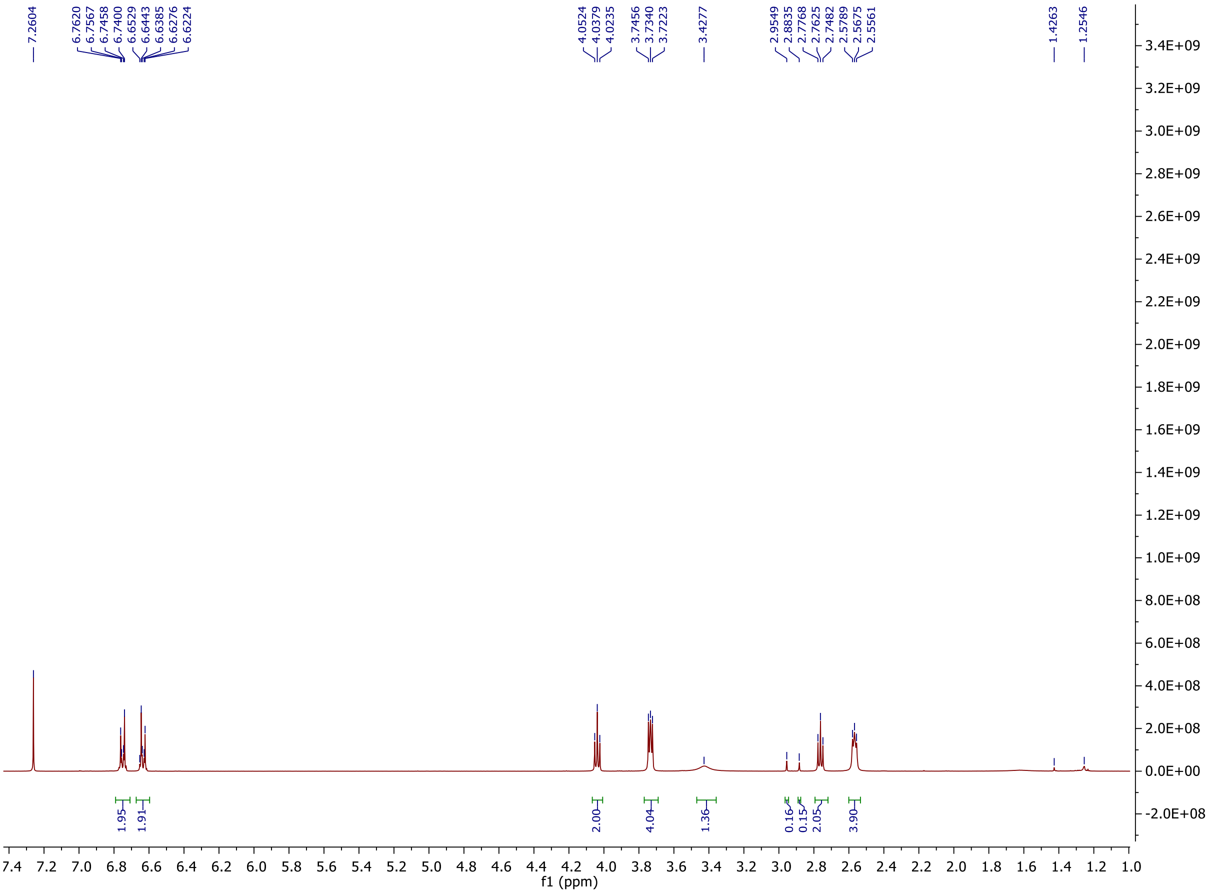

Figure S4. ^{1}H NMR spectrum of 4-[2-(morpholin-4-yl)ethoxy]aniline (5).

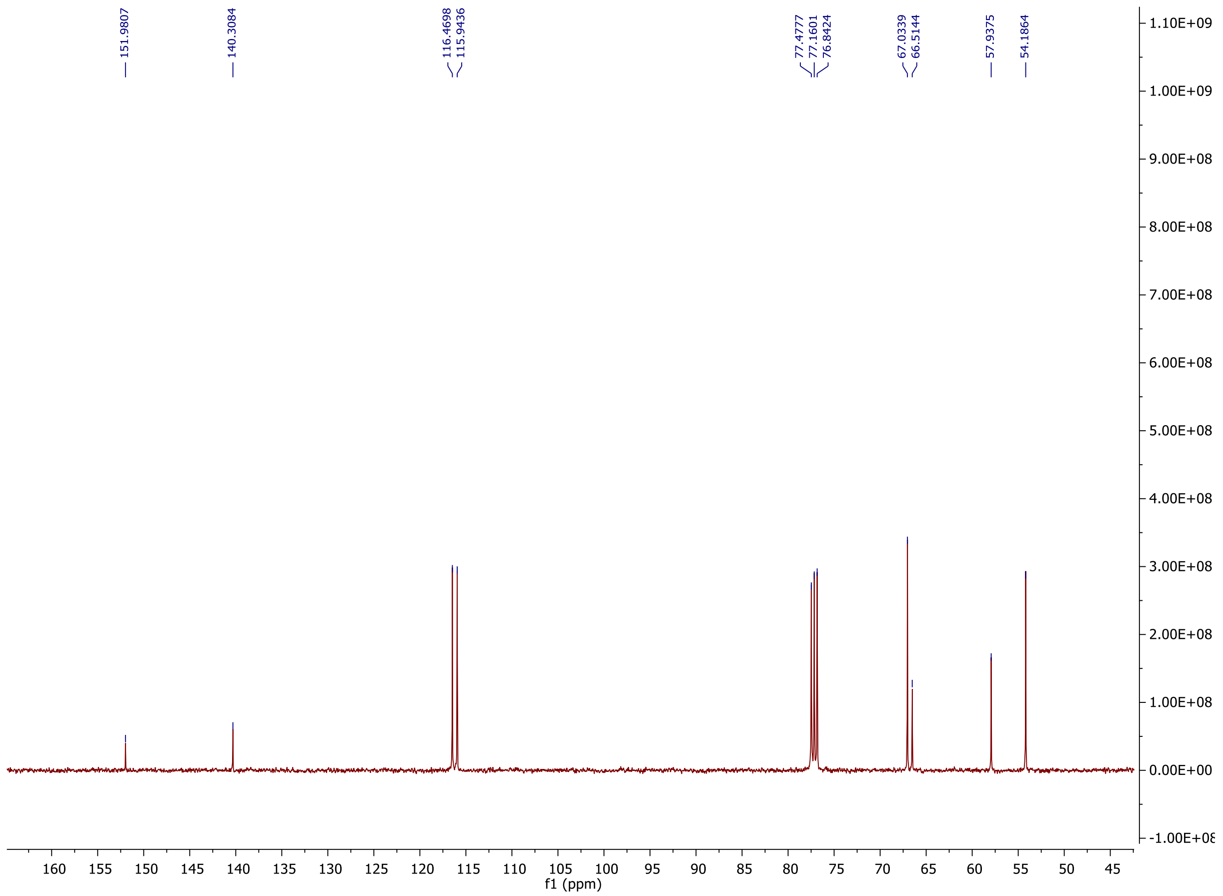

Figure S5. ^{13}C NMR spectrum of 4-[2-(morpholin-4-yl)ethoxy]aniline (5).

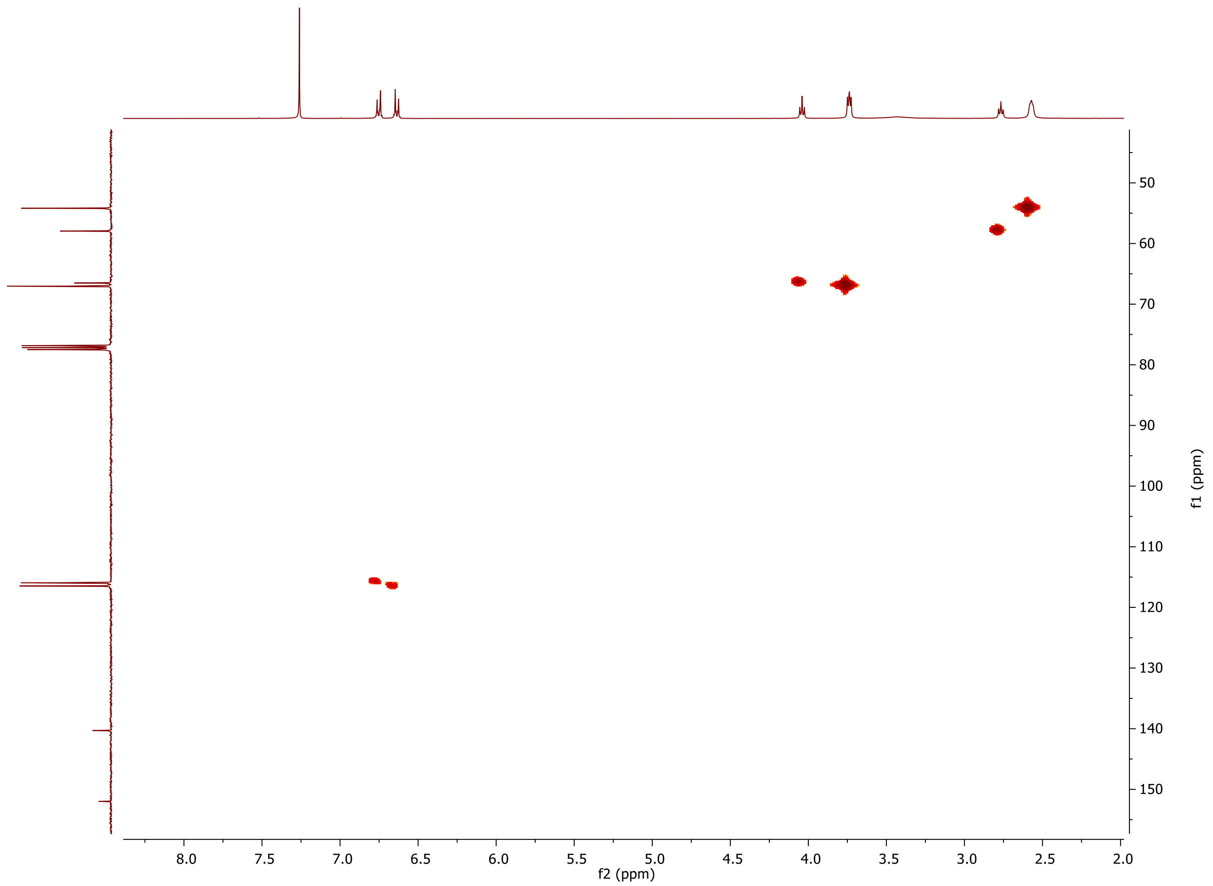

Figure S6. HSQC spectrum of 4-[2-(morpholin-4-yl)ethoxy]aniline (5).

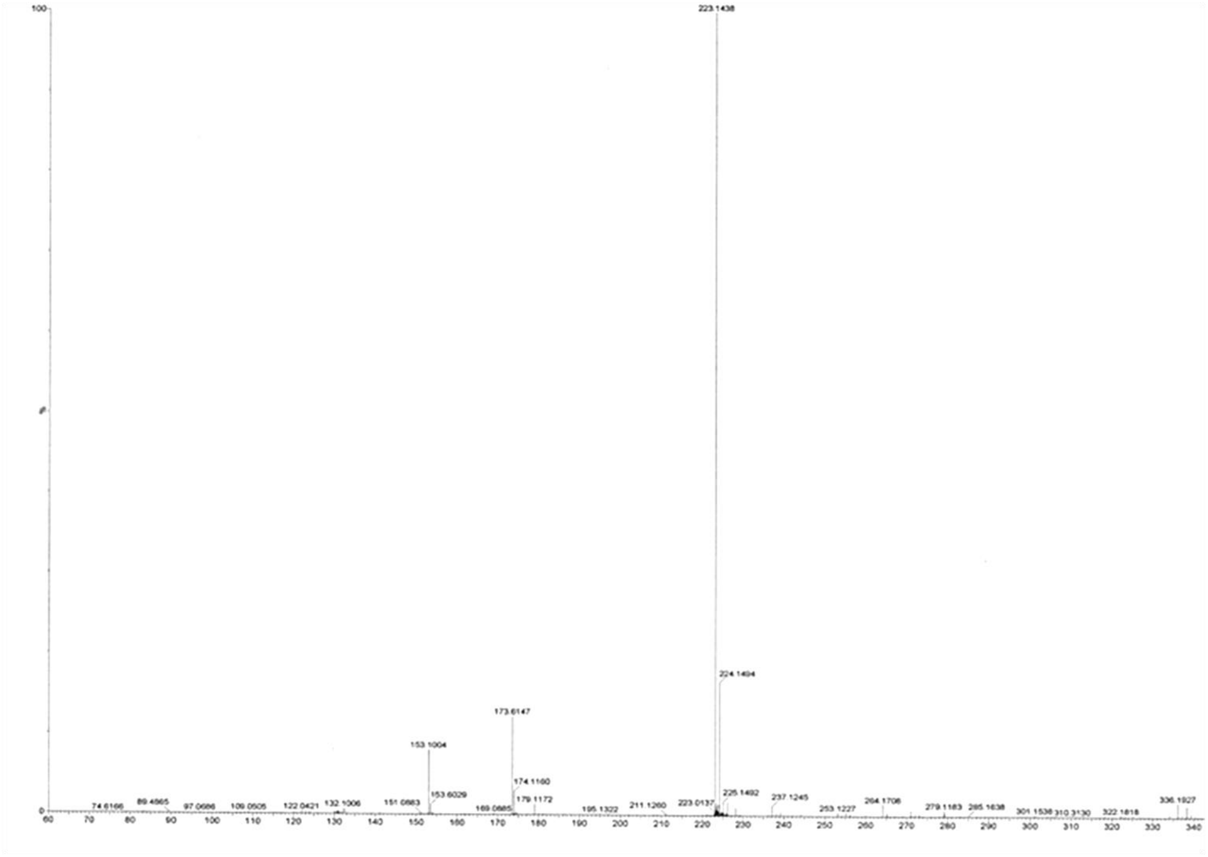

Figure S7. ESI(+)-MS of 4-[2-(morpholin-4-yl)ethoxy]aniline (5).

Synthesis of compound (6).

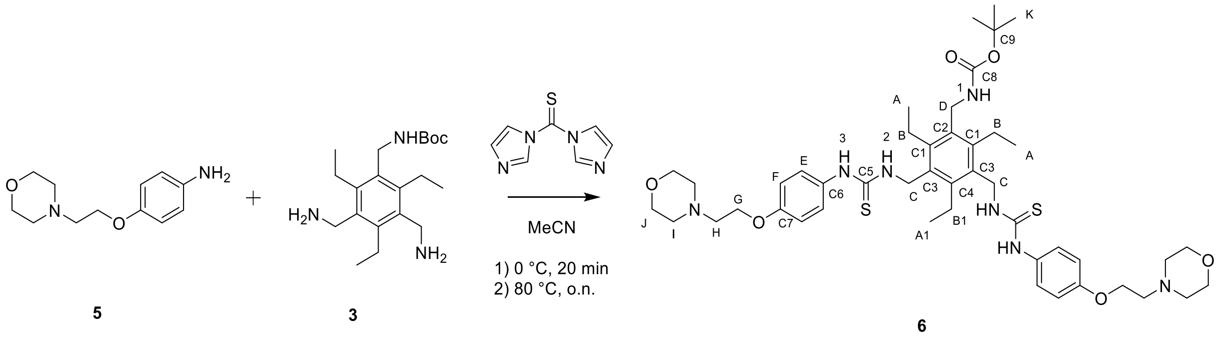

Di-amine **3** (332 mg, 0.95 mmol) in anhydrous MeCN (13.0 mL) was added dropwise to a stirring solution of 1,1'-thiocarbonyldiimidazole (342 mg, 1.92 mmol) in anhydrous MeCN (13.0 mL) cooled in an ice-water bath. The reaction mixture was stirred for 30 min in an ice-bath under nitrogen before amine **5** (427 mg, 1.92 mmol) in anhydrous MeCN (10.0 mL) was

added dropwise at a rapid rate. The reaction mixture was heated at reflux under nitrogen overnight. The reaction mixture was concentrated *in vacuo* and the residue was partitioned between $CHCl_3$ (50.0 mL) and H_2O (50.0 mL). The layers were separated and the aqueous layer back-extracted with $CHCl_3$ (2 × 40.0 mL). The combined organic layers were washed with water (3 × 80.0 mL). The resulting aqueous layer was further back-extracted with $CHCl_3$ (5 × 50.0 mL). The resulting combined organic layers were dried using Na_2SO_4 and following filtration under gravity concentrated *in vacuo*. Flash column chromatography eluting with $CHCl_3$/MeOH (9.8:0.2) followed by $CHCl_3$/MeOH (9.5:0.5) gave thiourea **6** as a pale orange solid foam (717 mg, 86%). TLC ($CHCl_3$/MeOH (9:6:0.4): R_f = 0.18; ^{1}H-NMR (400 MHz, $CDCl_3$) δ 1.07 p.p.m. (3H, br t, *J* 7.2, A1), 1.13 (6H, br t, *J* 7.4, A), 1.43 (9H, br s, K), 2.61-2.64 (12H, H and I), 2.84 (6H, br s, B and B1), 3.74-3.75 (8H, J), 4.13 (4H, m, G), 4.28 (2H, br s, D), 4.75 (4H, br s, C), 6.87 (4H, d, *J* 8.7, F), 7.09 (4H, d, *J* 8.7, E); ^{13}C-NMR (100 MHz, MeOD) δ 16.8 p.p.m (A), 16.9 (A1), 24.0 (B and B1), 28.8 (K), 39.7 (C), 44.4 (D), 55.1 (I), 58.7 (H), 66.6 (G), 67.6 (J), 80.3 (C9), 116.0 (F), 128.0 (E), 132.8 (C2), 133.9 (C1 and C3), 145.5 (C4), 145.8 (C7), 158.0 (C8), 158.4 (C6), 182.4 (C5); m/z (electrospray) 878.4654 (MH^+, 40%), 439.7334 (MH_2^{2+}, 100 %) Found: MH^+, 878.4654. $C_{46}H_{68}N_7O_6S_2$ requires 878.4673; Δ = -2.2ppm);

Spectra for compound (6).

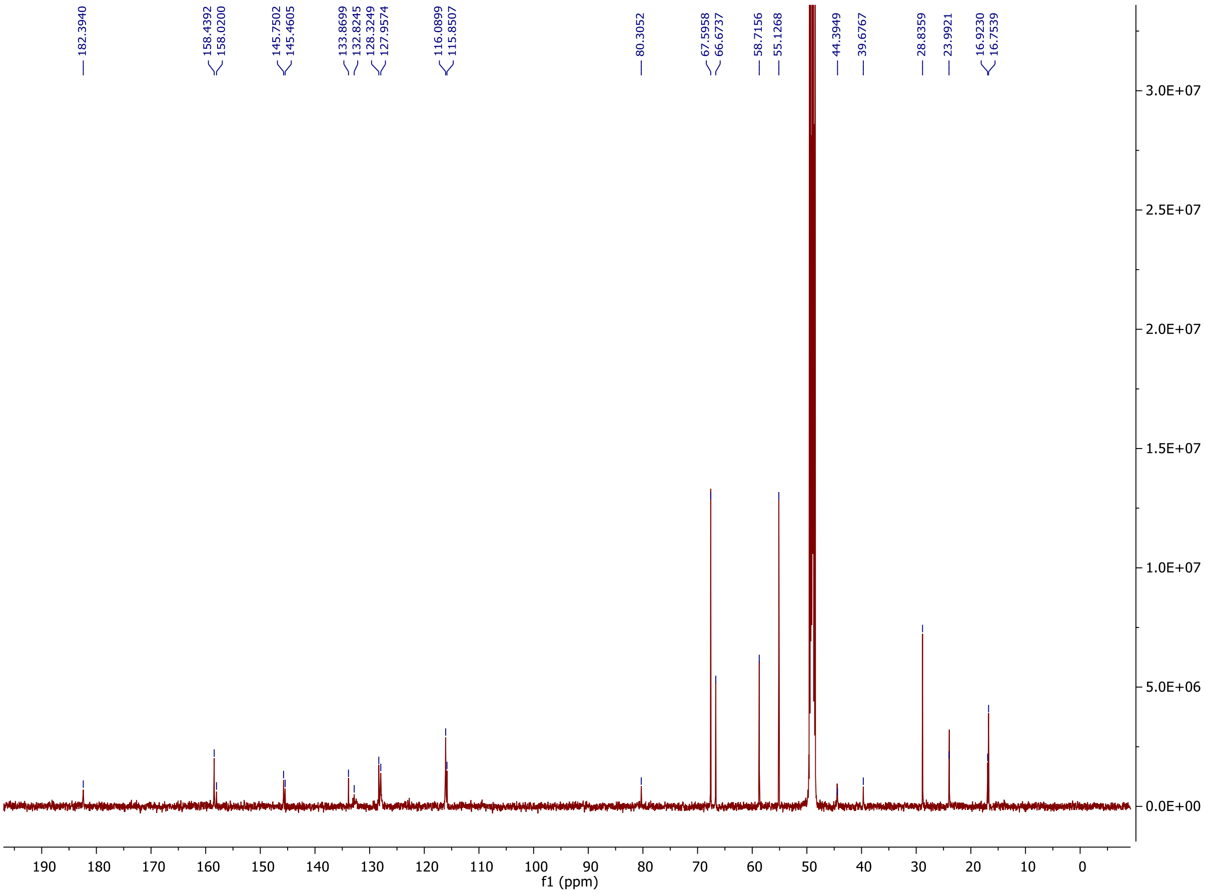

Figure S8. ^{13}C NMR spectrum of compound (6).

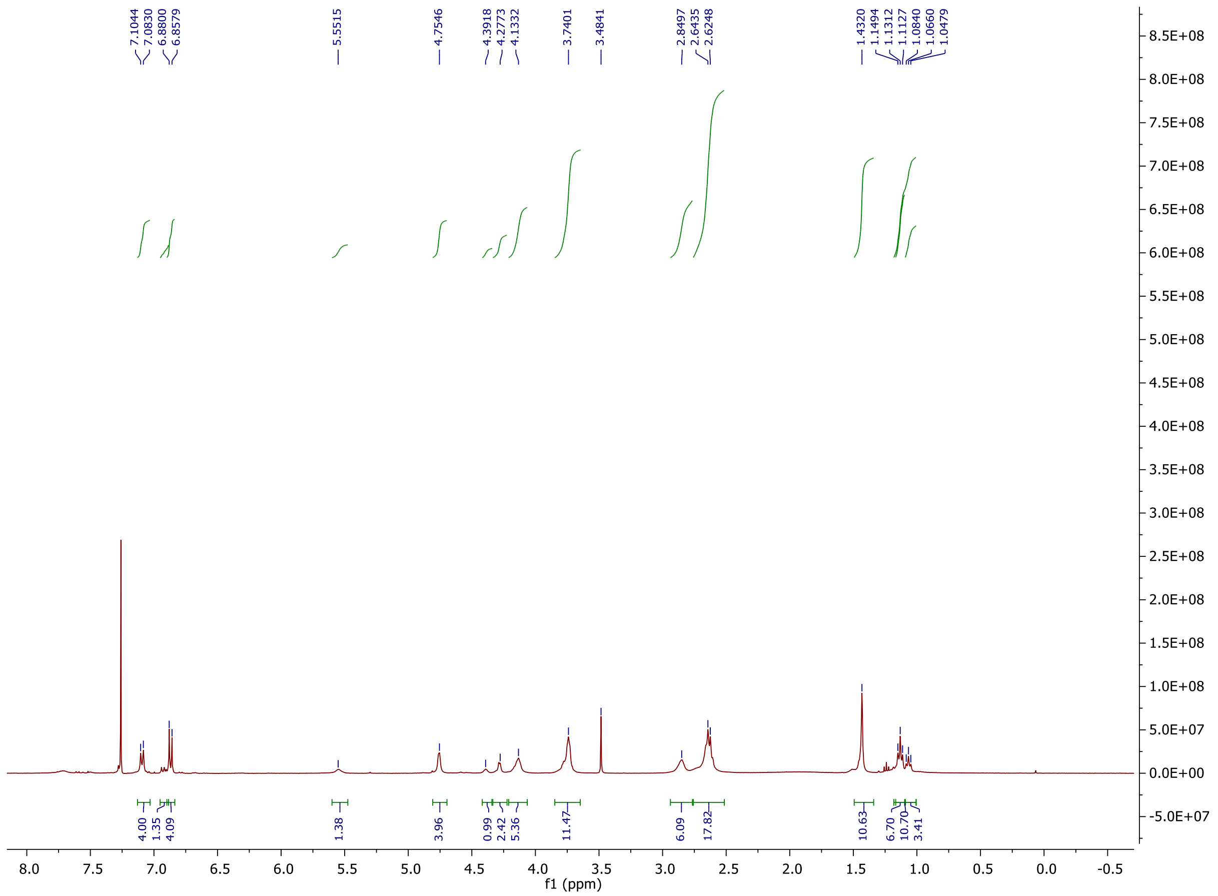

Figure S9. ^{1}H NMR spectrum of compound (6).

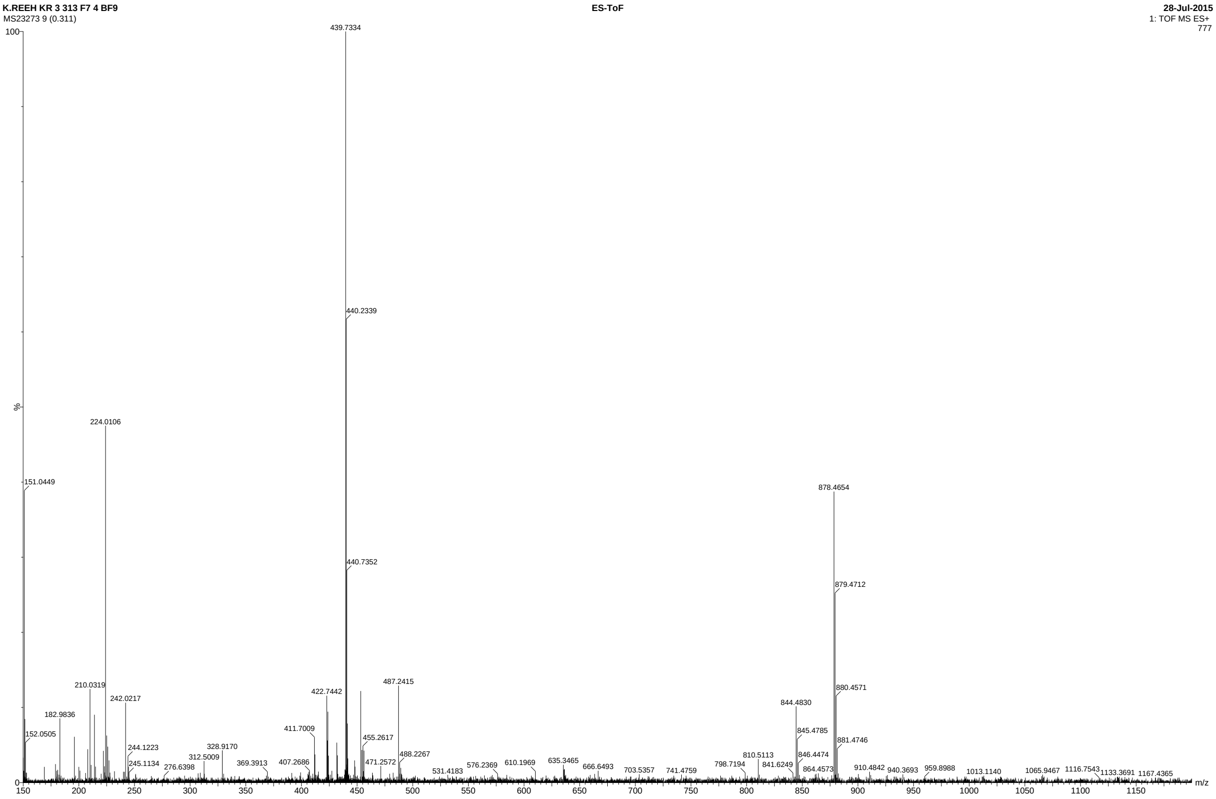

Figure S10. MS spectrum of compound (6).

Preparation of compound (7).

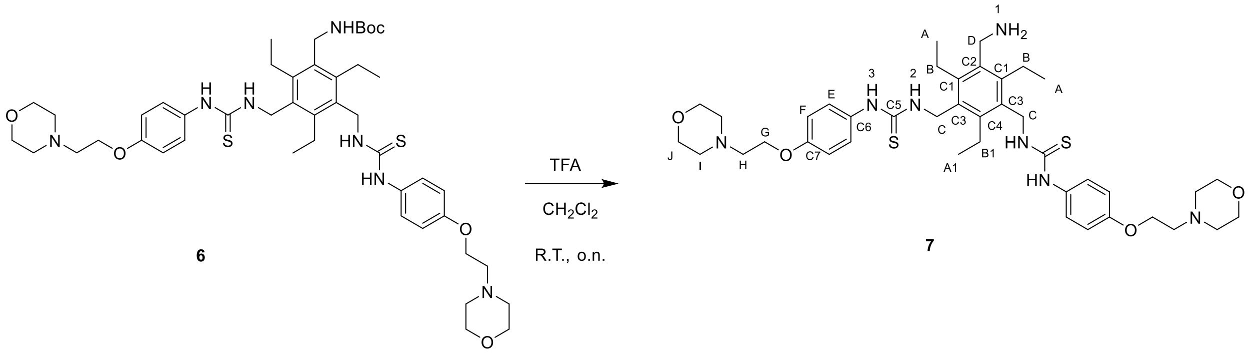

To a stirred solution of Boc-protected amine **6** (694 mg, 0.790 mmol) in anhydrous CH_2Cl_2 (18.00 mL) was added TFA (4.42 mL, 57.7 mmol). The reaction mixture was stirred at room temperature overnight. The reaction mixture was concentrated *in vacuo*. The residue was partitioned between $CHCl_3$ (20 mL) and 2 M NaOH (20.0 mL). The layers were separated and the organic layer further washed with 2 M NaOH (20.0 mL) and H_2O (20.0 mL). The aqueous layer was back-extracted with $CHCl_3$ (3 × 40.0 mL) followed by a 3 % MeOH in $CHCl_3$ solution (3 × 40.0 mL). The combined organic layers were dried using Na_2SO_4 and filtered under gravity. Flash column chromatography eluting with $CHCl_3$/MeOH (9.5:0.5), then

$CHCl_3$/MeOH/7N NH_3 in MeOH (9.45:0.5:0.05) gave amine **7** as a white solid foam (497 mg, 81 %). TLC ($CHCl_3$/7N NH_3 in MeOH (9:95:0.05)): $R_f = 0.16$; ^{1}H-NMR (400 MHz, MeOD) δ 1.18 p.p.m. (9H, t, *J* 7.1, A and A1), 2.57 (8H, m, I), 2.76 (10H, m, H, B and B1), 3.69 (8H, m, J), 3.86 (2H, s, D), 4.11 (4H, t, *J* 5.5, G), 4.74 (4H, br s, C), 6.91 (4H, d, *J* 8.8, F), 7.22 (4H, d, *J* 8.8, E); ^{13}C-NMR (100 MHz, MeOD) δ 16.9 p.p.m (A and A1), 23.9 (B), 39.4 (D), 44.5 (C), 55.1 (I), 58.7 (H), 66.7 (G), 67.6 (J), 116.1 (F), 127.0 (E), 132.4-8 (C1 and C3), 137.7 (C2), 144.8 (C7), 145.4 (C4), 158.4 (C6), 182.4 (C5); m/z (electrospray) 778.4164 (MH^+, 100%), 389.7068.2176 MH_2^{2+}, 40 %) Found: MH^+, 778.4164. $C_{41}H_{60}N_7O_4S_2$ requires 778.4148; Δ = 2.1ppm).

Spectra for compound (7).

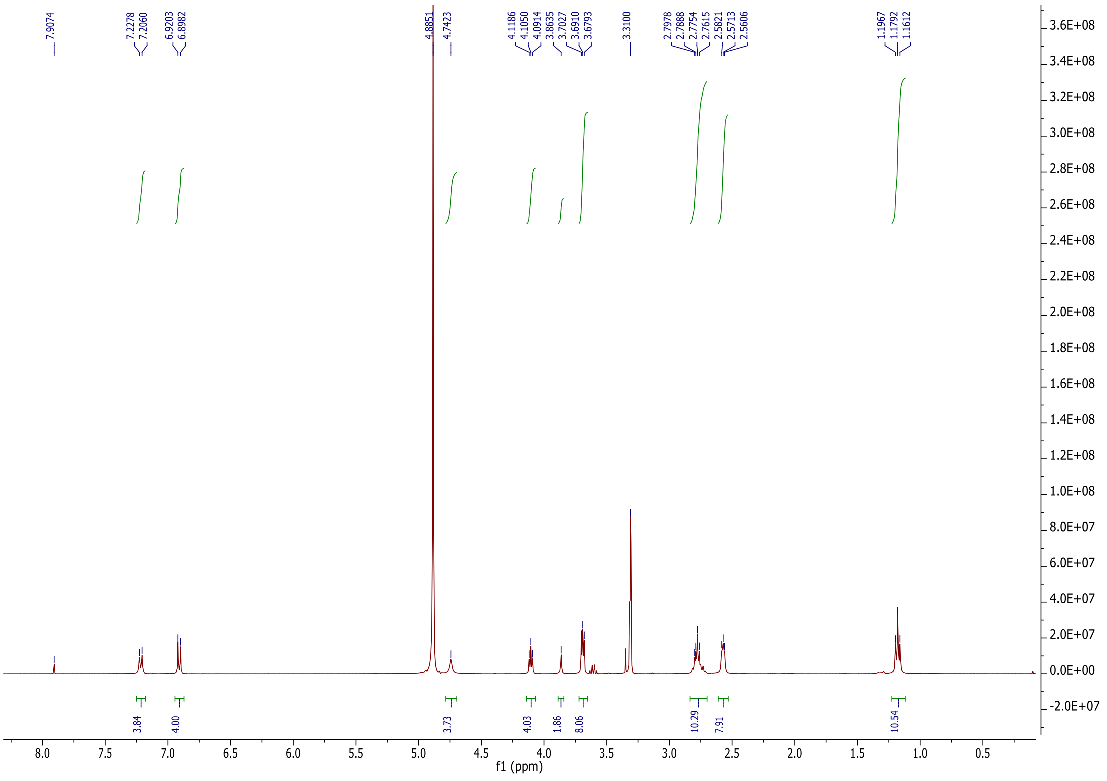

Figure S11. ^{1}H NMR spectrum of compound (7).

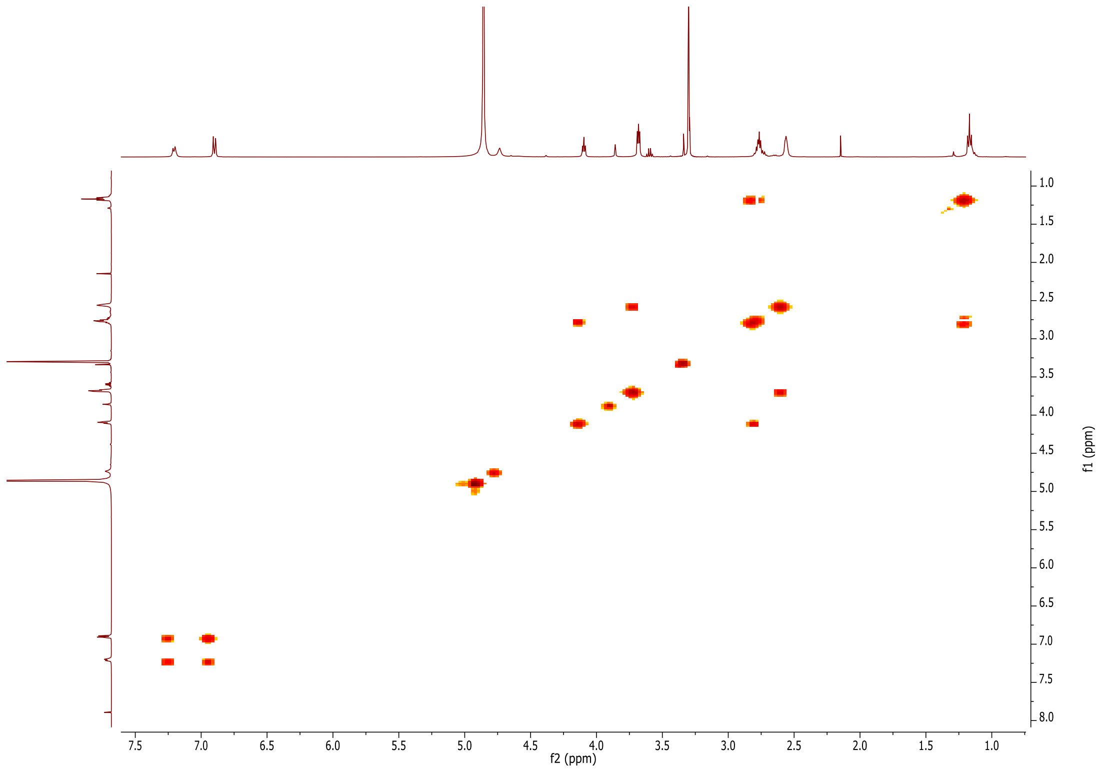

Figure S12. COSY spectrum of compound (7).

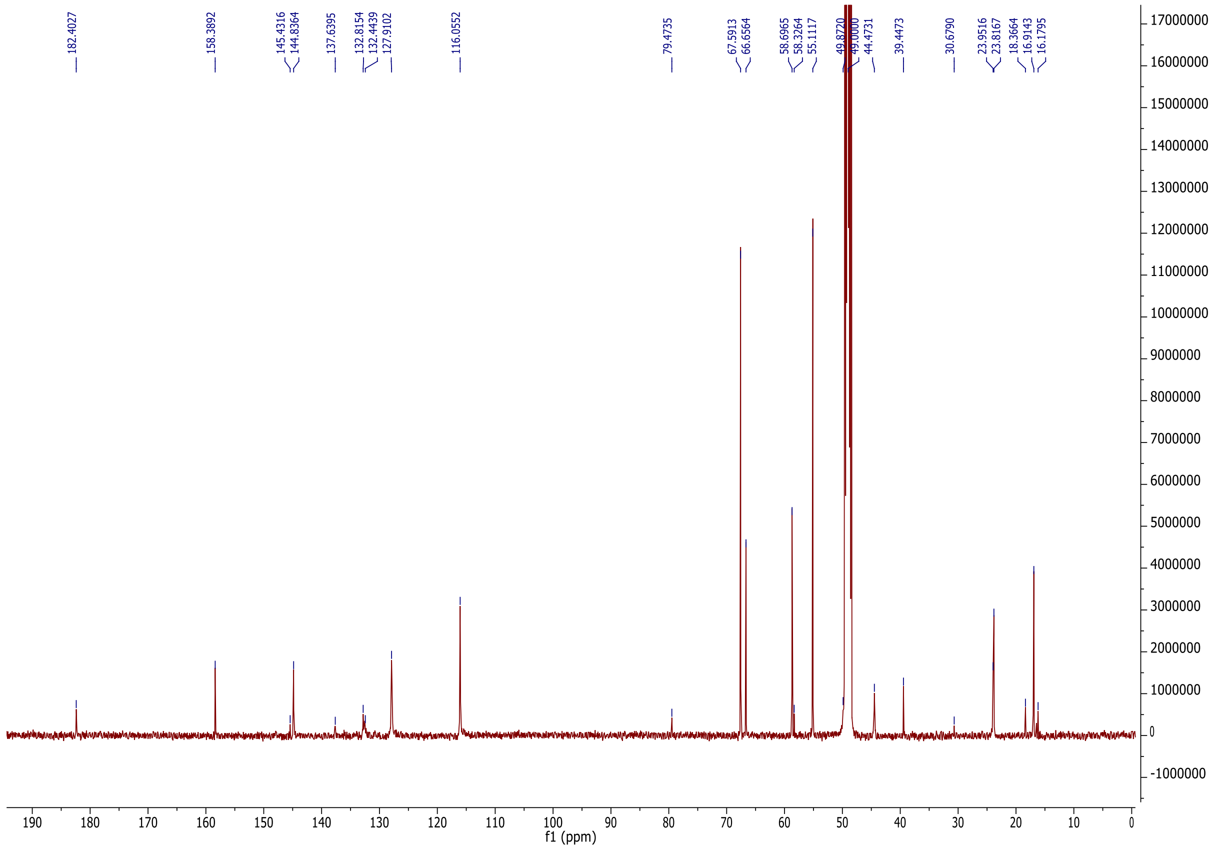

Figure S13. ^{13}C NMR spectrum of compound (7).

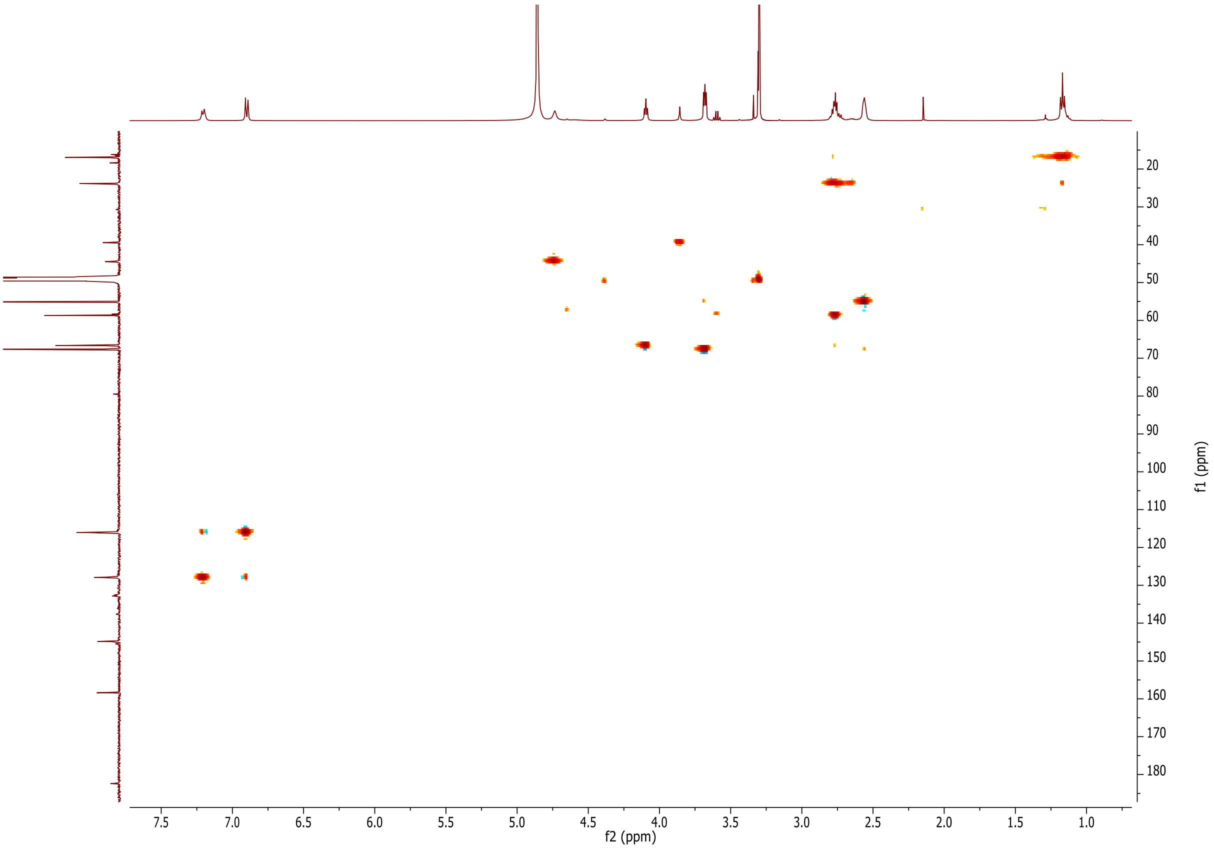

Figure S14. HSQC spectrum of compound (7).

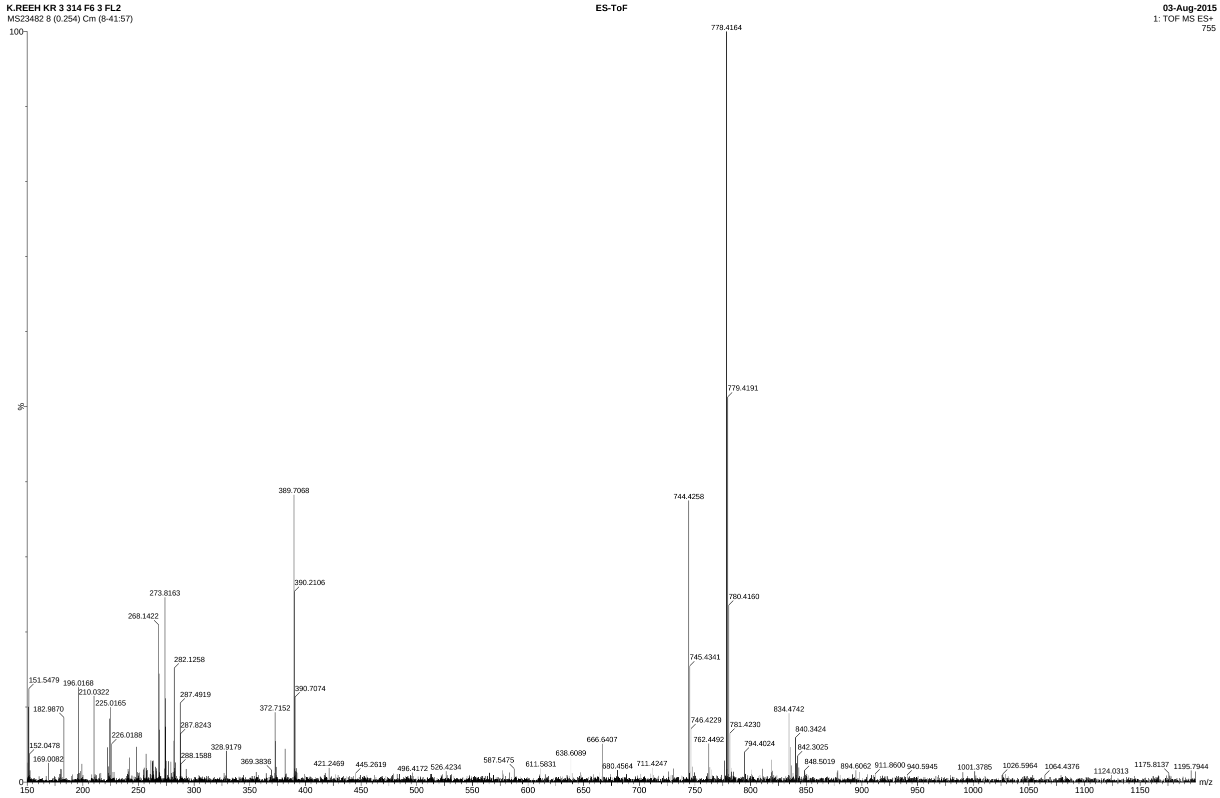

Elemental Composition Report

Single Mass Analysis

Tolerance = 10.0 PPM / DBE: min = -1.5, max = 50.0

Element prediction: Off

Number of isotope peaks used for i-FIT = 3

Monoisotopic Mass, Even Electron Ions

237 formula(e) evaluated with 1 results within limits (all results (up to 1000) for each mass)

Elements Used:

C: 41-41 H: 0-200 N: 0-10 O: 0-10 Na: 0-1 S: 2-2

Minimum:				-1.5					
Maximum:		5.0	10.0	50.0					
Mass	Calc. Mass	mDa		PPM		DBE	i-FIT	i-FIT (Norm)	Formula
778.4164	778.4148	1.6	2.1	15.5	45.0		0.0		C41 H60 N7 O4 S2

Figure S15. MS spectrum of compound (7).

Synthesis of compound (8).

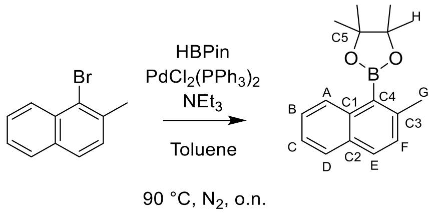

8

To a solution of 1-bromo-2-methylnaphthalene (0.200 mL, 1.283 mmol) in anhydrous toluene (10.0 mL) was added [Pd(PPh_3)$_2$$Cl_2$] (45.0 mg, 1.924 mmol; 5 mol%), NEt_3 (0.890 mL, 6.42 mmol) followed by HBpin (0.279 mL, 1.92 mmol). The solution was stirred under nitrogen at 90 °C for 19 hr. The reaction mixture was allowed to cool to room temperature and concentrated *in vacuo*. The residue was taken up in EtOAc (15 mL), filtered and the filtrate partitioned with water (15 mL). The layers were separated and the aqueous phase extracted with EtOAc (2 × 15 mL). The combined organic extracts were washed with water (2 × 30 mL), subsequently dried using Na_2SO_4 and following filtration under gravity concentrated *in vacuo*. Flash column chromatography eluting with hexane then hexane/EtOAc (9.8:0.2) gave boronate ester **8** as white needles (300 mg, 87 %). TLC (hexane/ EtOAc, 9.8:0.2 v/v): R_f = 0.2; υ_{max} /cm^{-1} 3051 (aromatic C-H stretch), 2975 and 2929 (methyl C-H stretch), 1298 (B-O stretch), 1260 (CH_3 bend), 1129 (C-O stretch); ^{1}H-NMR (400 MHz, $CDCl_3$) δ 1.47 p.p.m. (12H, s, H), 2.68 (3H, s, G), 7.33 (1H, d, *J* 8.4, F), 7.42 (1H, m, C), 7.50 (1H, m, B), 7.80 (2H, m, D and E), 8.17 (1H, d, *J* 8.4, A); ^{13}C-NMR (100 MHz, $CDCl_3$) δ 22.7 p.p.m. (G), 25.2 (H), 84.1 (C5), 124.7 (C), 126.1 (B), 127.6 (A), 128.2 (D), 128.6 (F), 129.7 (E) , 131.5 (C3), 136.7 (C4), 141.5 (C1 and C2); m/z (EI) Found: M^+, 268.1626. $C_{17}H_{21}$11BO_2 requires M^+, 268.1635; Δ = -3.4ppm).

Spectra for compound (8).

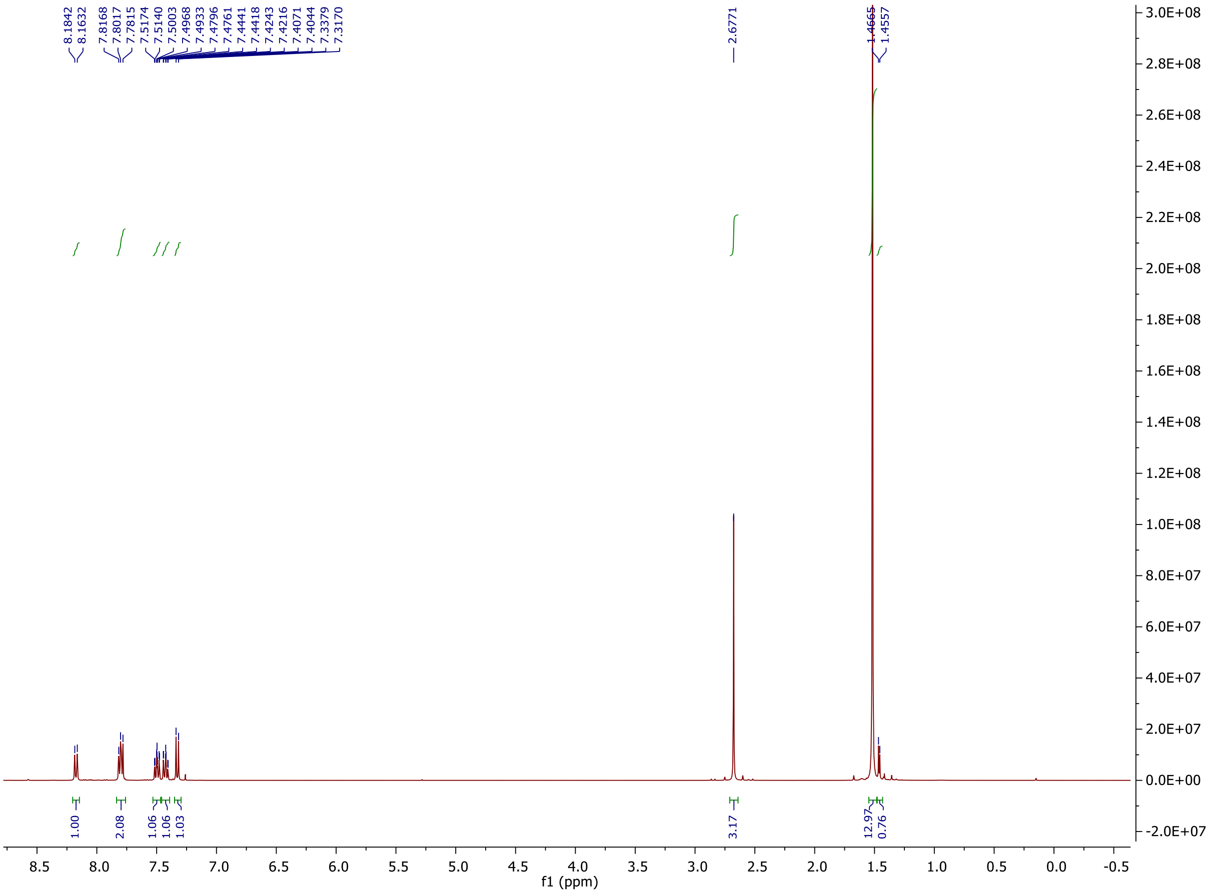

Figure S16. ¹H NMR spectrum of compound (8).

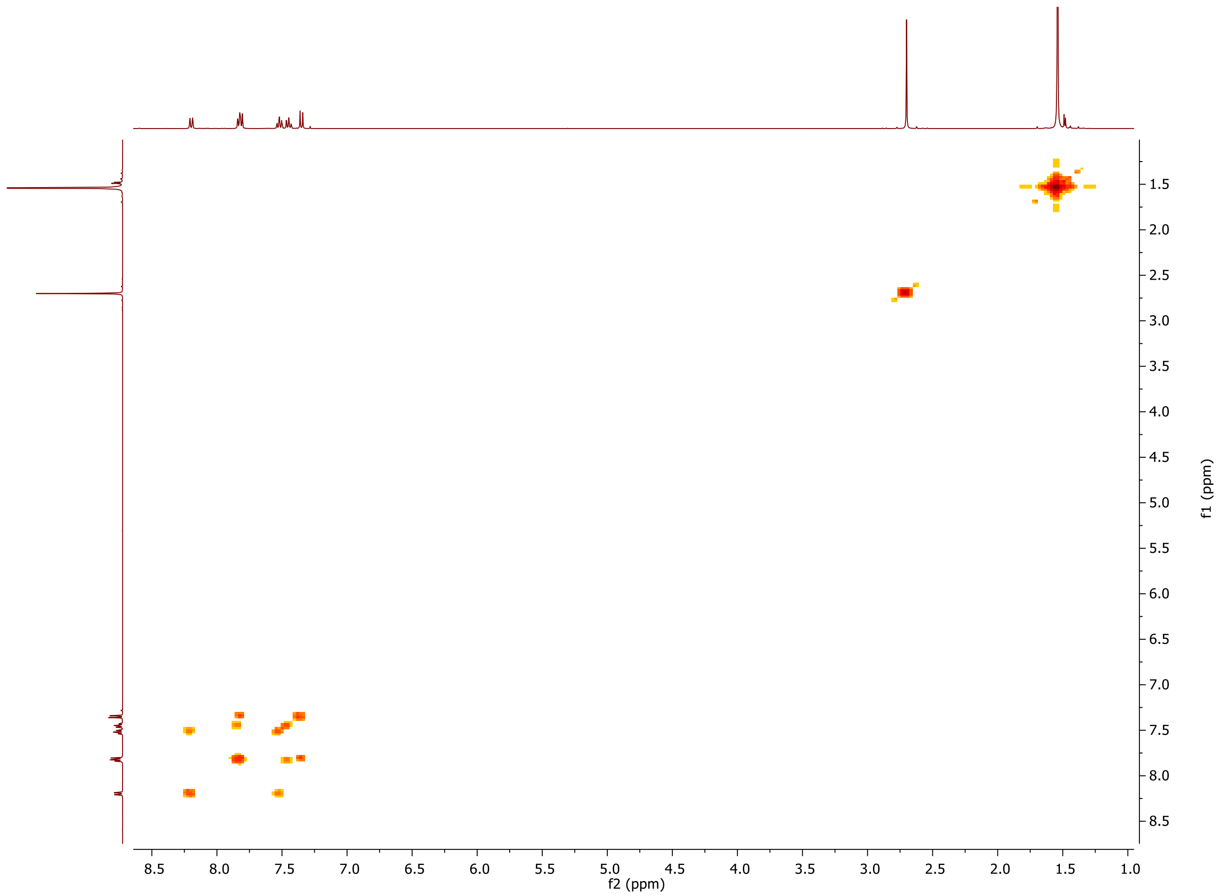

Figure S17. COSY spectrum of compound (8).

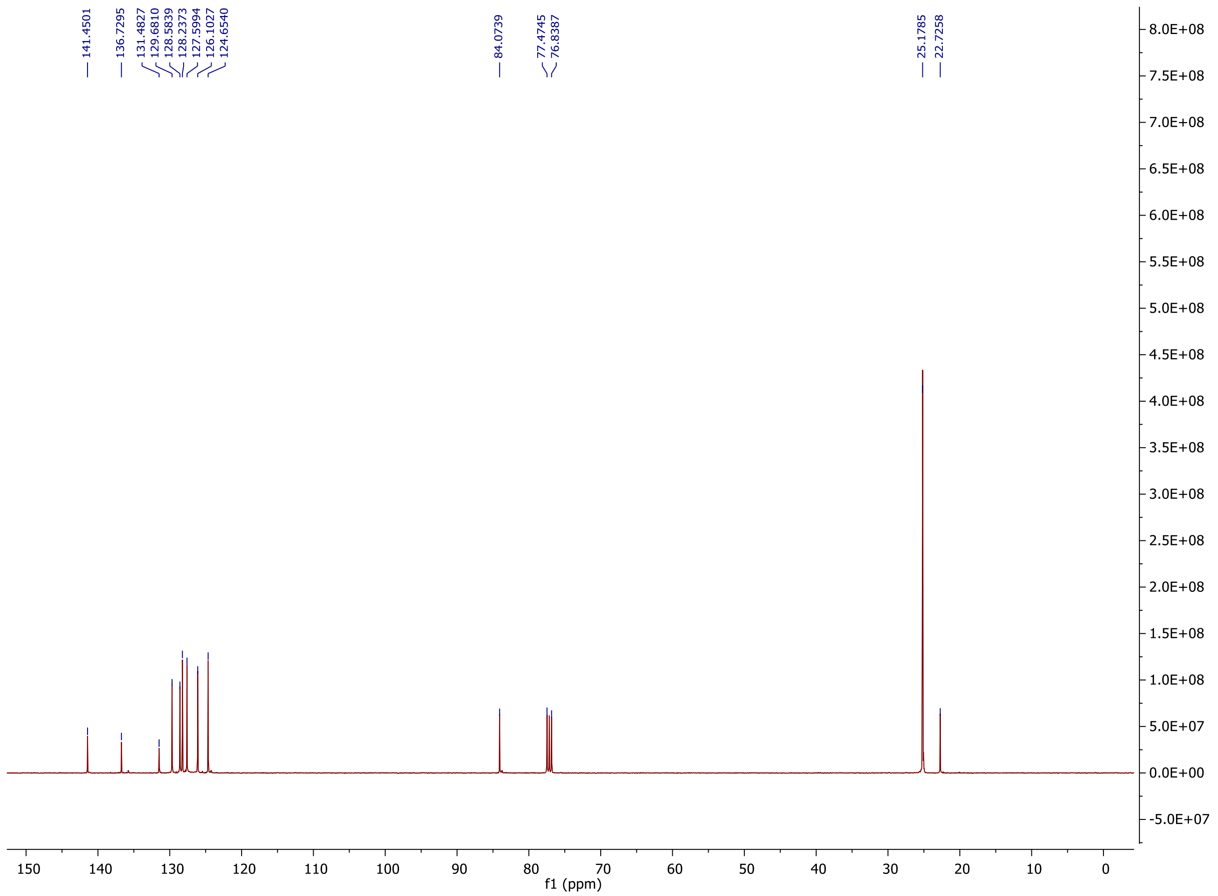

Figure S18. ^{13}C NMR spectrum of compound (8).

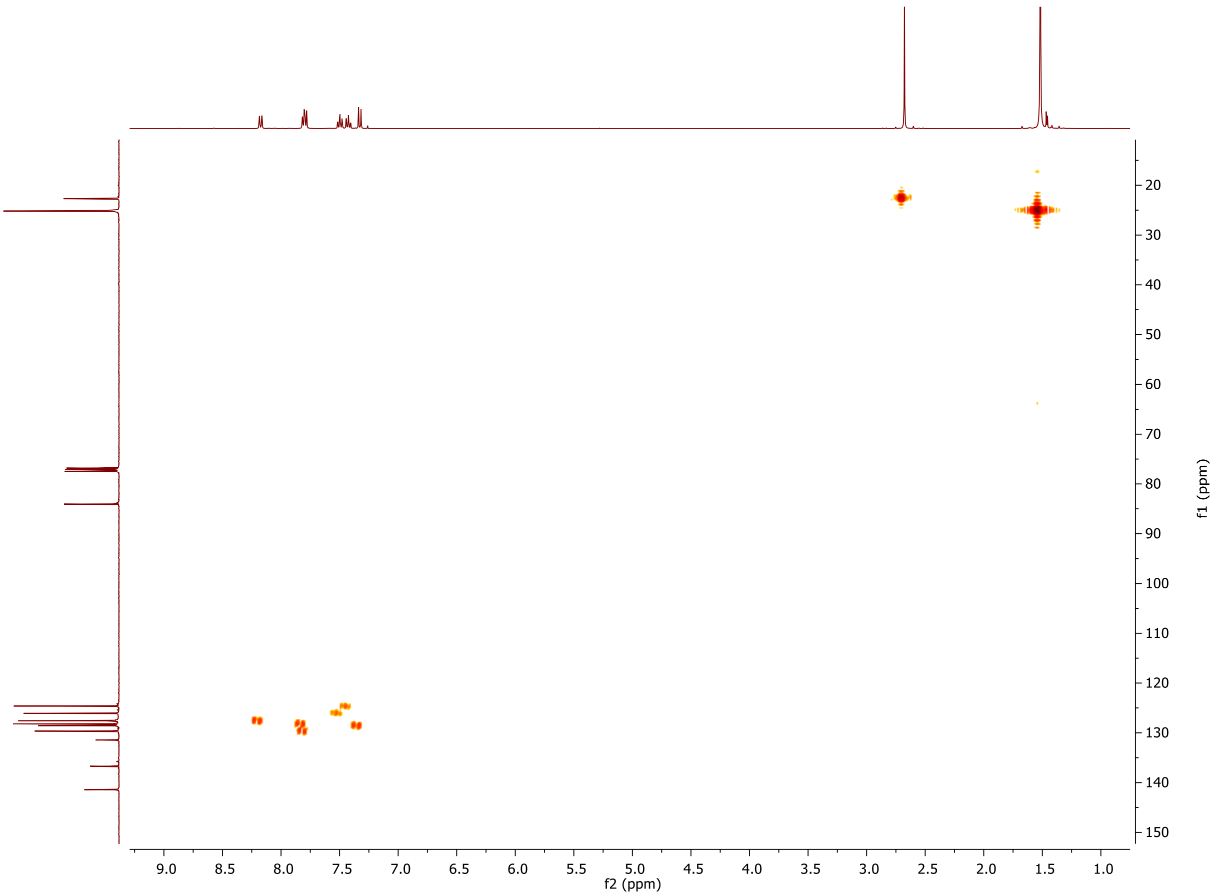

Figure S19. HSQC spectrum of compound (8).

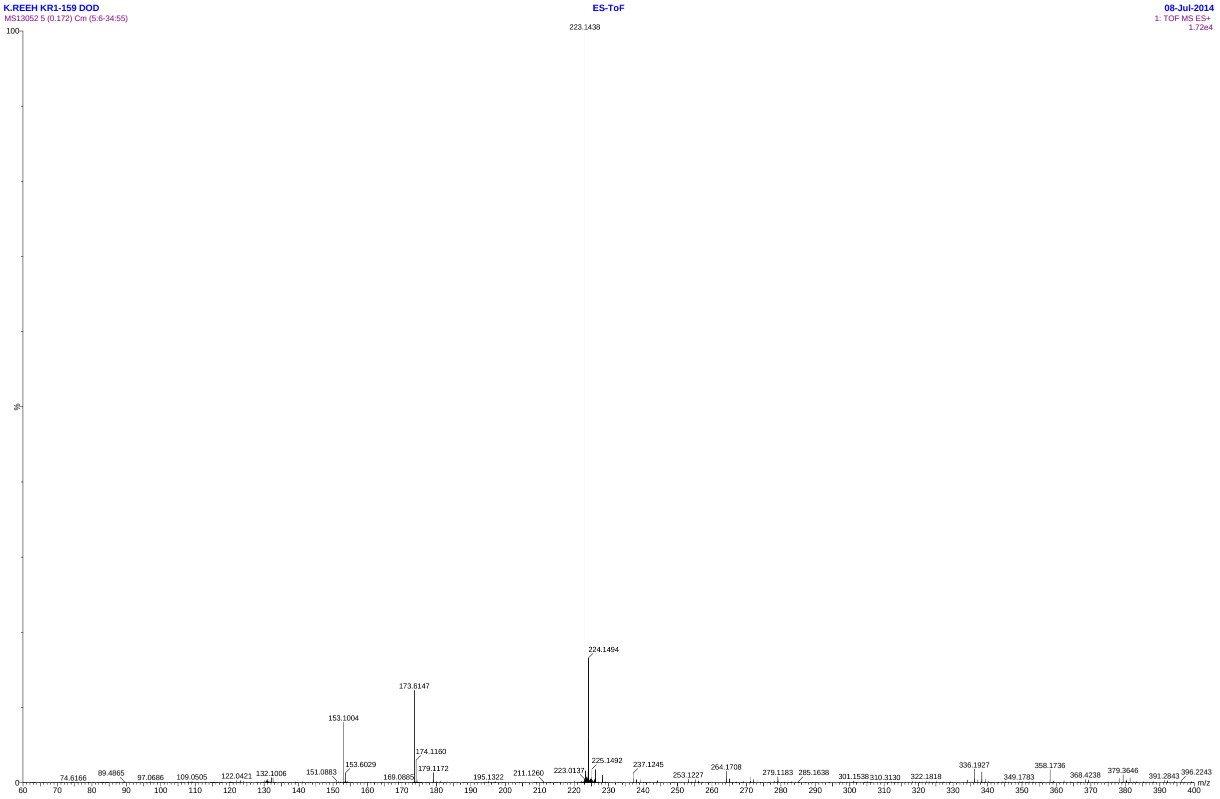

Figure S20. MS spectrum of compound (8).

Synthesis of compound (9).

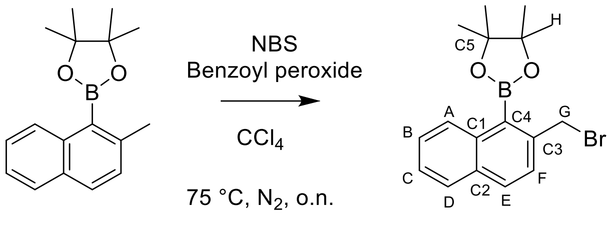

8 9

A solution of compound **8** (1.042 g, 3.89 mmol), N-bromosuccinimide (830 mg, 4.66 mmol) and benzoyl peroxide (113 mg, 0.466 mmol, 12mol %) in anhydrous CCl_4 (10.0 mL) under nitrogen was stirred at reflux overnight. The reaction mixture was allowed to cool to room temperature and concentrated *in vacuo*. Flash column chromatography of the residue eluting with hexane then hexane/EtOAc (9.9:0.1) gave compound **9** as off-white needles (1.05 g, 81 %). TLC (hexane/ EtOAc, 9.5:0.5 v/v): $R_f = 0.3$; υ_{max} /cm^{-1} 3071 (aromatic C-H stretch), 2976 and 2928 (methyl C-H stretch), 1294 (B-O stretch), 1259 (aliphatic CH bend), 1129 (C-O stretch), 662 (C-Br stretch); ^{1}H-NMR (400 MHz, $CDCl_3$) δ 1.54 p.p.m. (12H, s, H), 4.99 (2H, s, G), 7.50 (3H, m, B, C and F), 7.80 (1H, br d, *J* 9.2, D), 7.87 (1H, br d, *J* 8.8, E), 8.32 (1H, d, *J* 8.4, A); ^{13}C-NMR (100 MHz, $CDCl_3$) δ 25.2 p.p.m. (H), 34.2 (G), 84.1 (C5), 126.3 (C), 126.7 (F), 127.8 (B), 128.4 (D), 128.6 (A), 130.9 (E) , 132.8 (C3), 136.7 (C4), 141.8 (C1 and C2); m/z (EI) Found: M^+, 346.0742. $C_{17}H_{20}11BO_2Br$ requires M^+, 346.0740; Δ = 0.6ppm).

Spectra for compound (9).

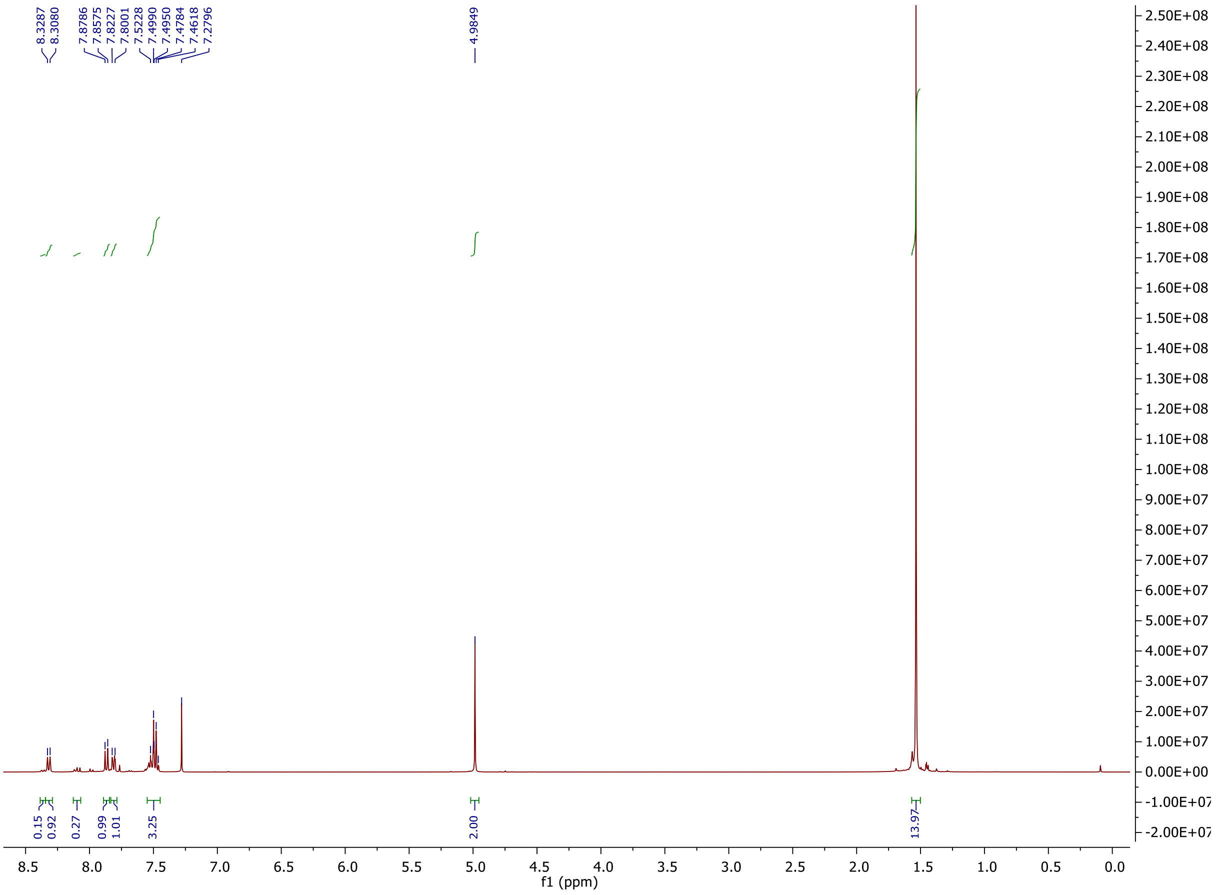

Figure S21. ^{1}H NMR spectrum of compound (9).

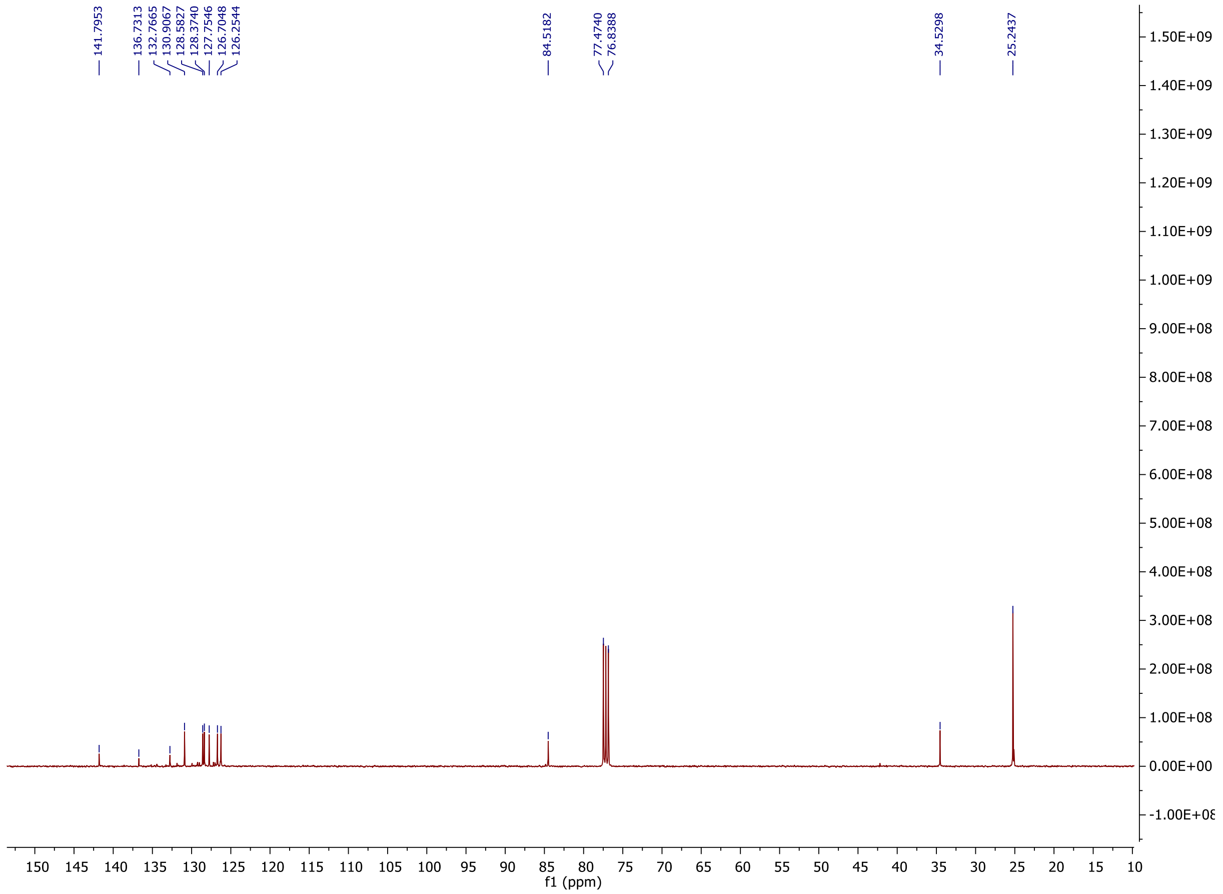

Figure S22. ^{13}**C NMR spectrum of compound (9).**

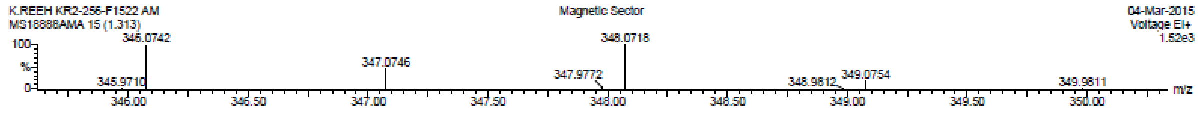

Figure S23. MS spectrum of compound (9).

Synthesis of compound (10).

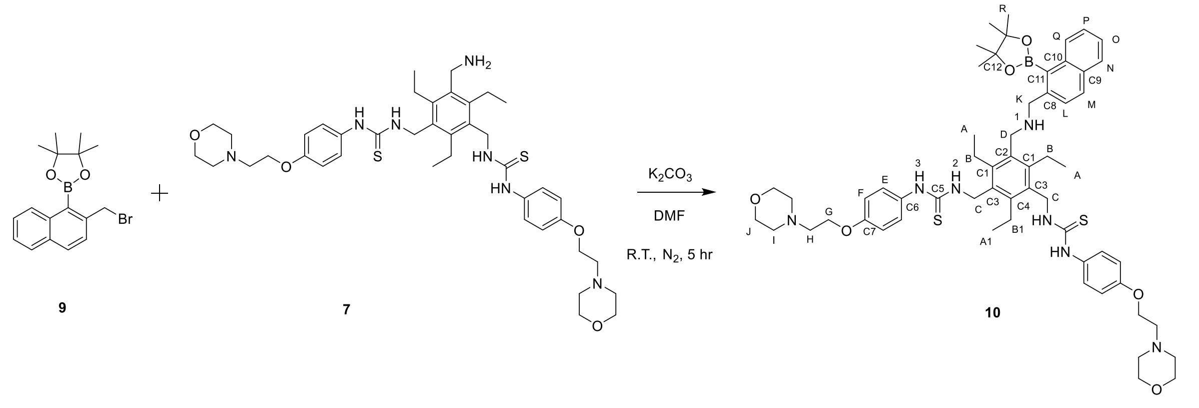

A solution of amine **7** (474 mg, 0.609 mmol) and K_2CO_3 (51.0 mg, 0.366 mmol) in anhydrous DMF (6.00 mL) was stirred under nitrogen for 30 min before a solution of bromine **9** (106 mg, 0.305 mmol) in anhydrous DMF (2.50 mL) was added dropwise over 25 min. The reaction mixture was stirred under nitrogen at room temperature for 6 hr. The reaction mixture was concentrated *in vacuo* and the residue was partitioned between $CHCl_3$ (15 mL) and brine (15.0 mL). The layers were separated and the aqueous layer back-extracted with $CHCl_3$ (2 × 10.0 mL). The organic layers further were washed with brine (3 × 30.0 mL). The resulting aqueous layer was further back-extracted with $CHCl_3$ (3 × 40.0 mL) followed by a 3 % MeOH in $CHCl_3$ solution (3 × 40.0 mL). The resulting combined organic layers were dried using Na_2SO_4 and following filtration under gravity concentrated *in vacuo*. Flash column chromatography eluting with $CHCl_3$/MeOH (9.85:0.15), then $CHCl_3$/MeOH (9.5:0.5) gave boronate ester **10** as an off-white foam solid (149 mg, 48 %). TLC ($CHCl_3$/MeOH (9:98:0.02)): R_f = 0.29; ^{1}H-NMR (400 MHz, MeOD) δ 0.90 p.p.m. (6H, br t, *J* 7.2, A), 1.22 (3H, m, A1), 1.46 (12H, s, R), 2.56 (12H, m, I and B), 2.78 (6H, m, H and B), 3.68 (8H, br t, *J* 4.6, J), 3.78 (2H, br s, K), 3.97 (2H, br s, D), 4.12 (4H, br t, *J* 5.4, G), 4.80 (4H, br s, C), 6.95 (4H, d, *J* 8.8, F), 7.13 (1H, br d, *J* 8.0, L), 7.24 (4H, br d, *J* 8.8, E), 7.44 (1H, br t, *J* 6.8, O), 7.50 (1H, br t, *J* 6.8, P), 7.81 (1H, d, *J* 8.0, M), 7.86 (1H, d, *J* 8.0, N), 8.6 (1H, d, *J* 8.1, Q); ^{13}C-NMR (100 MHz, MeOD) δ 16.5 (A), 16.8 (A1), 24.2 (B and B1), 26.6 (R), 43.8 (D), 44.4 (C), 49.0 (K), 55.1 (I), 58.7 (H), 66.7 (G), 67.6 (J), 81.8 (C12), 116.1 (F), 123.0 (L), 126.2 (O), 126.4 (P), 128.0 (E), 128.6 (Q), 129.6 (N), 130.1 (C11), 130.4 (M), 132.5 (C3), 133.3 (C1), 135.0 (C8), 136.8 (C9 and C10), 140.0 (C2), 146.7 (C4), 147.1 (C7), 158.5 (C6), 182. 4 (C5); m/z (electrospray) 1044.5657 (MH^+, 100%), 522.7809 MH_2^{2+}, 70 %) Found: MH^+, 1044.5657. $C_{58}H_{79}N_7O_6S_2$11B requires 1044.5626; Δ = 3.0ppm).

Spectra for compound (10).

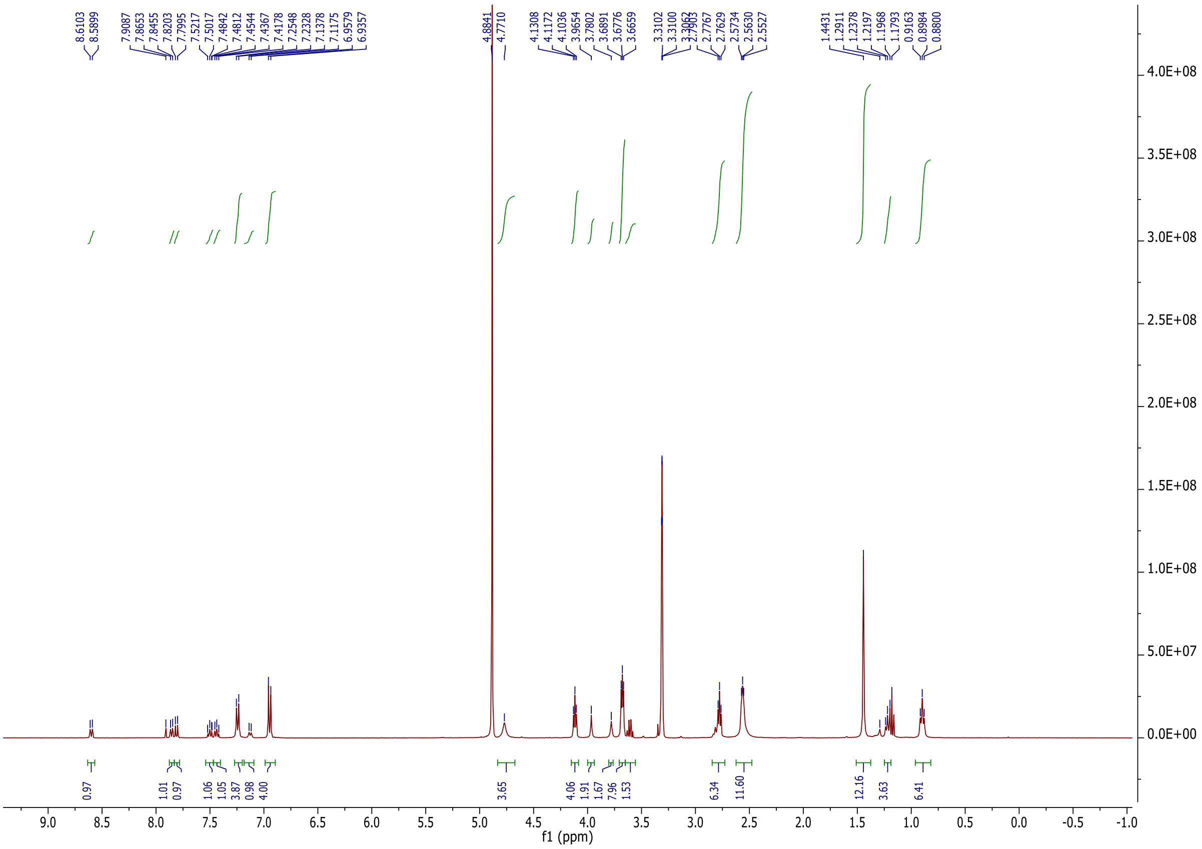

Figure S24. ¹H NMR spectrum of compound (10).

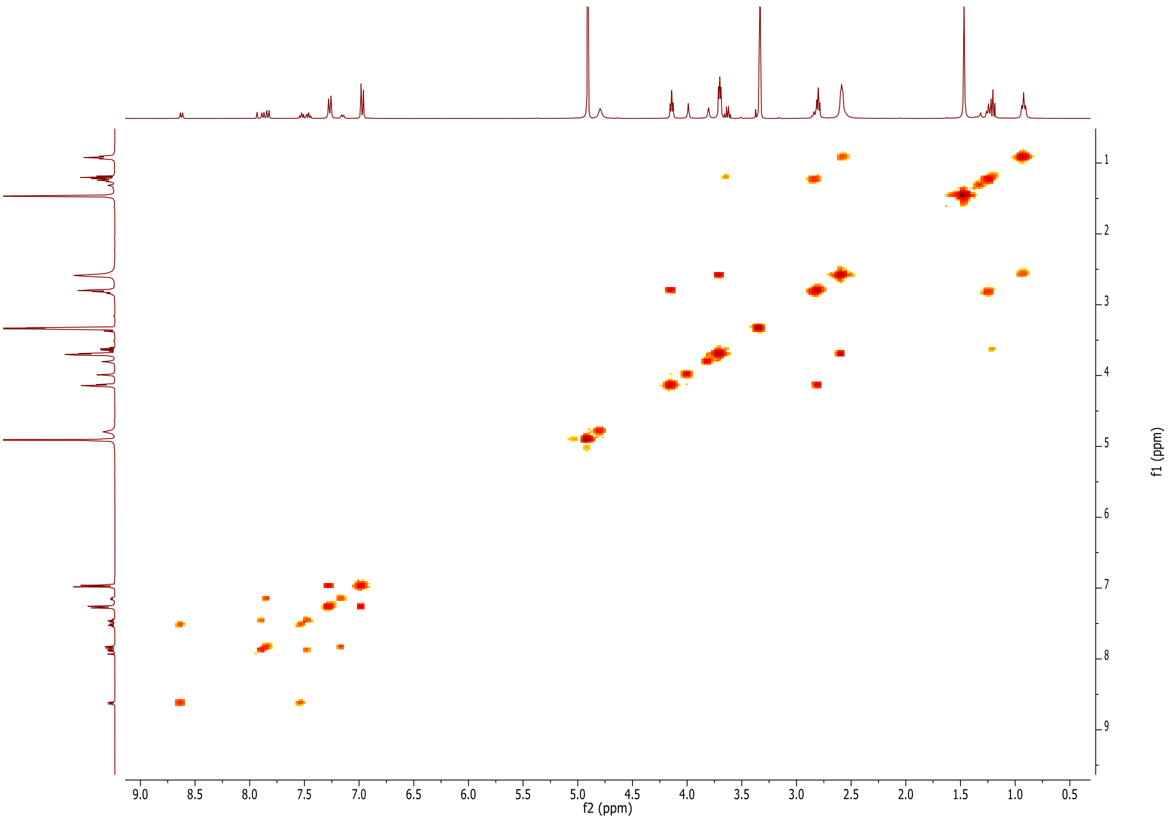

Figure S25. COSY spectrum of compound (10).

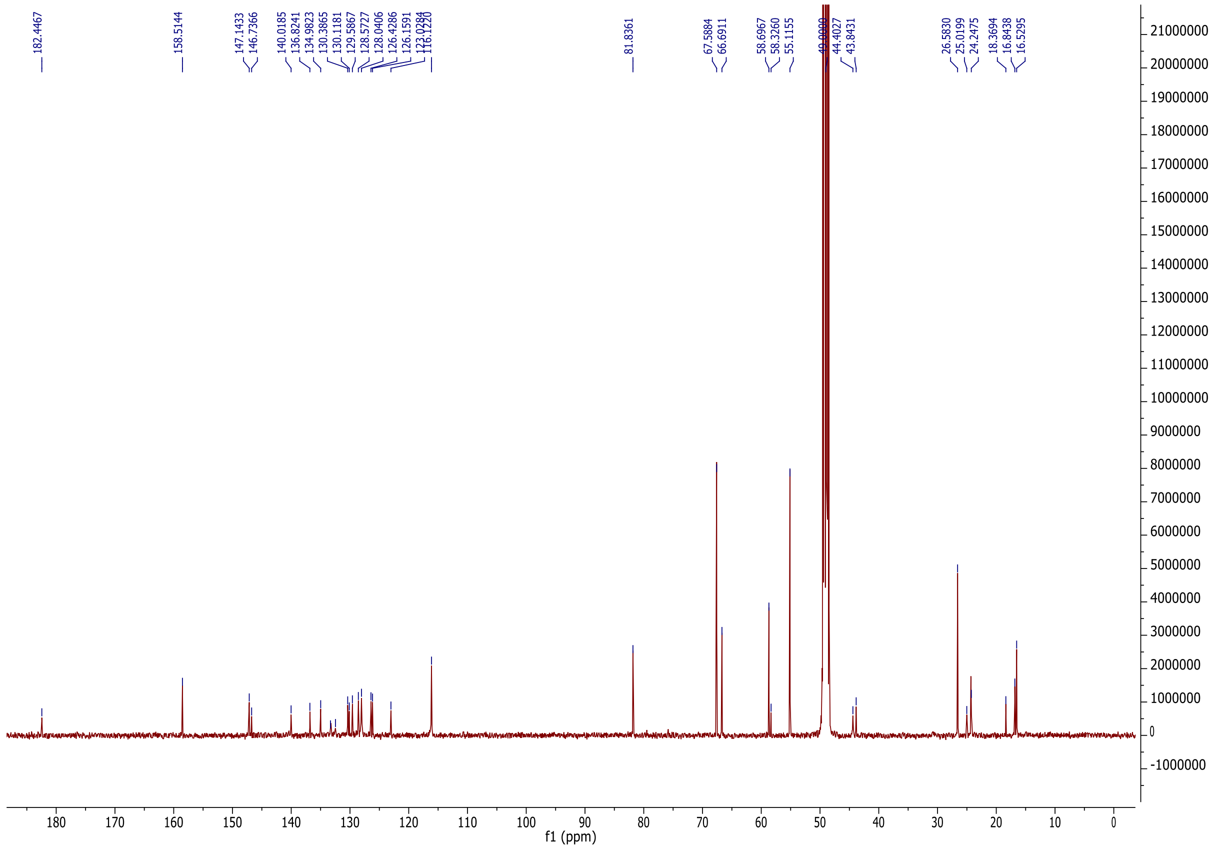

Figure S26. **^{13}C NMR spectrum of compound (10).**

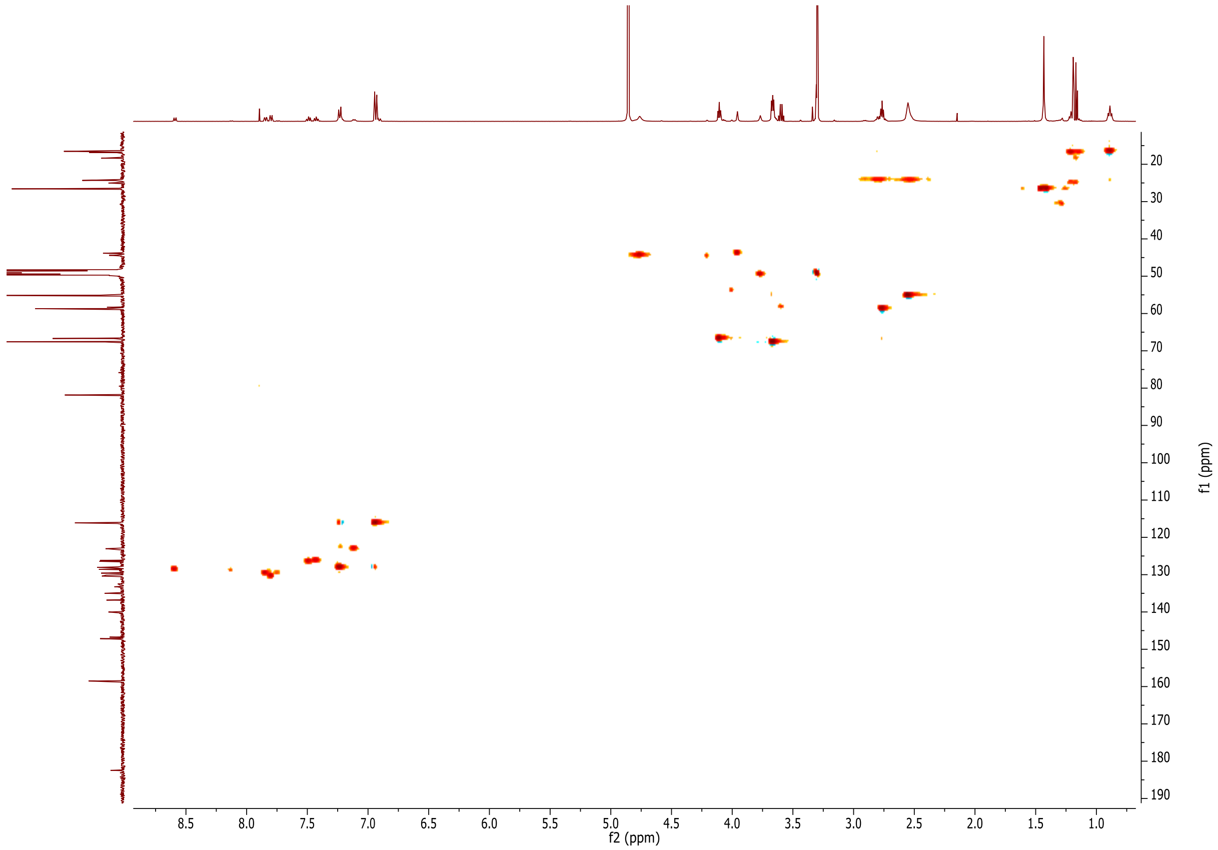

Figure S27. HSQC spectrum of compound (10).

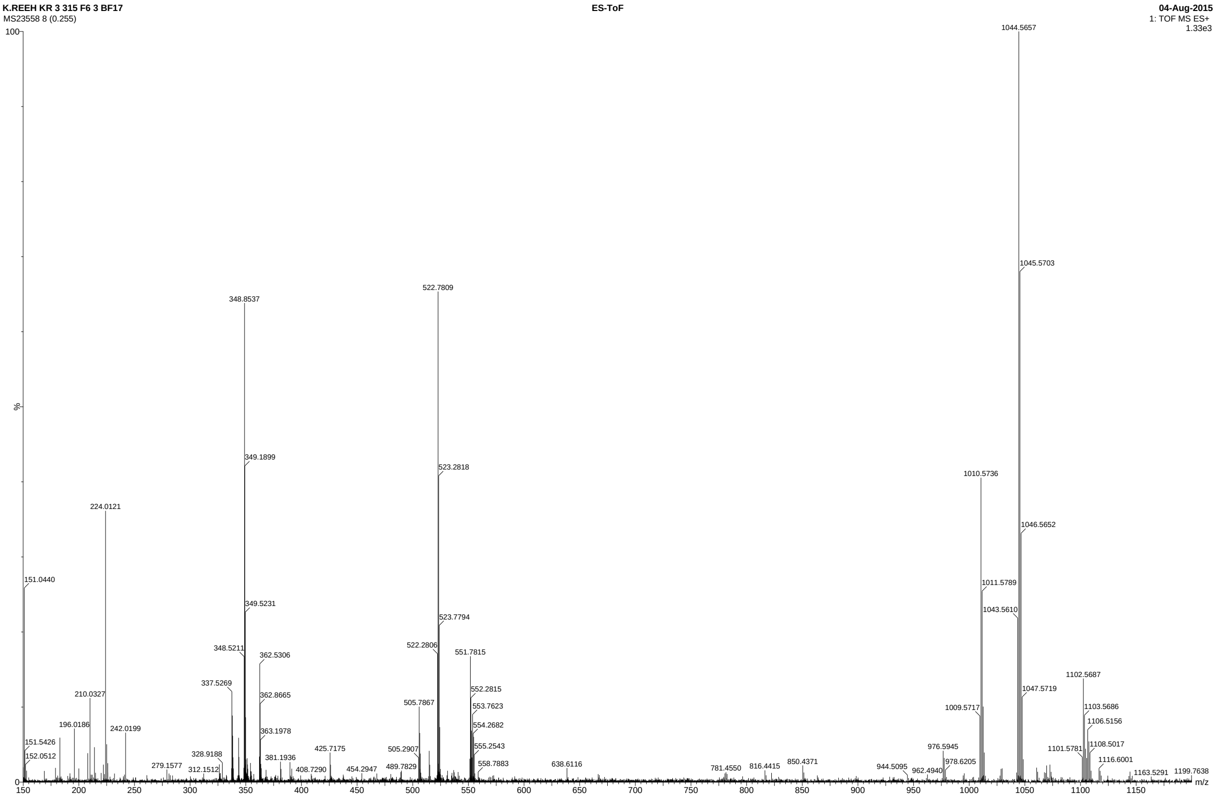

Elemental Composition Report

Single Mass Analysis

Tolerance = 10.0 PPM / DBE: min = -1.5, max = 50.0

Element prediction: Off

Number of isotope peaks used for i-FIT = 3

Monoisotopic Mass, Even Electron Ions

695 formula(e) evaluated with 3 results within limits (all results (up to 1000) for each mass)

Elements Used:

C: 58-58 H: 0-200 N: 0-10 O: 0-15 Na: 0-1 S: 2-2 11B: 0-1

Minimum:				-1.5						
Maximum:		5.0	10.0	50.0						
Mass	Calc. Mass	mDa		PPM		DBE	i-FIT	i-FIT (Norm)		Formula
1044.5657	1044.5626	3.1	3.0	23.5	27.9		1.0		C58 H79 N7 O6 S2 11B	

Figure S28. MS spectrum of compound (10).

Synthesis of receptor (11).

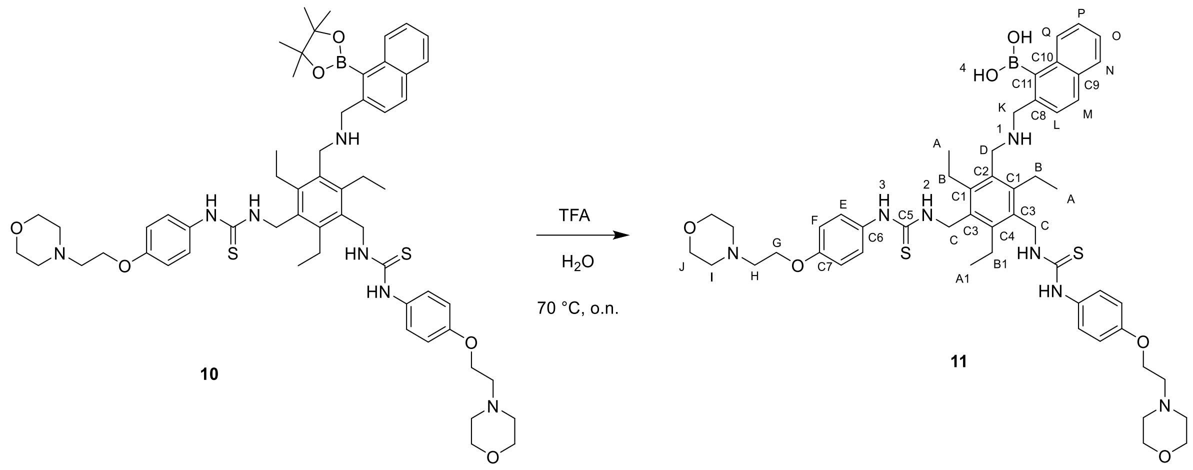

Boronate ester **10** (120 mg, 0.115 mmol) was stirred in a 70 % TFA : H_2O solution (5.80 mL) at 68 °C for 24 hr. The solvent was removed *in vacuo*. The residue was taken up in $CHCl_3$ (10 mL) and washed with 2 M NaOH (2 × 10.0 mL). The aqueous layer was further extracted with $CHCl_3$ (5 × 7.00 mL). The combined organic layers were concentrated *in vacuo*. The resulting precipitate was washed several times with methanol and isolated the centrifuge. This process gave boronic acid **11** as an off-white powder (65 mg, 59 %). ^{1}H-NMR (400 MHz, DMSO) δ 1.13 p.p.m. (6H, br t, *J* 7.2, A), 1.20 (3H, br t, *J* 7.3, A1), 2.49 (8H, m, J), 2.69 (4H, t, *J* 5.8, H), 2.78 (2H, br d, *J* 7.3, B1), 2.97 (6H, m, B), 3.21 (1H, s, N1H), 2.59 (8H, m, J), 3.92 (2H, brs, D), 4.07 (4H, t, *J* 5.8, G), 4.72-4.74 (6H, C and K), 6.86 (4H, d, *J* 9.0, 7.06 (2H, m, N2H), 7.34 (4H, d, *J* 9.0, E), 7.39 (1H, br d, 8.3, L), 7.44 (1H, m, O), 7.51 (1H< m, P), 7.80 (1H, m, M), 7.87 (1H, m, N), 8.55 (1H, d, *J* 8.2, Q), 9.04 (2H, br s, $B(OH)_2$); ^{13}C-NMR (100 MHz, DMSO) δ 15.5 p.p.m. (A), 15.6 (A1), 21.7 (B), 22.1 (B1), 41.2 (K), 42.2 (C), 51.3 (D), 53.1 (I), 56.6 (H), 65.7 (J and G), 114.2 (F), 120.6 (L), 124.2 (O), 124.6 (E), 125.1 (P), 126.4 (Q), 127.4 (N), 128.1 (M), 131.4 (C1), 131.6 (C3), 132.2 (C2 and C4), 132.8 (C8), 134.0 (C11) 142.8 (C9), 143.6 (C10), 148.8 (C7), 155.2 (C6), 180.7 (C5); LCMS trace 100 %; m/z (electrospray) 962.4866 (MH^+, 100%), 944.4828 (MH^+-OH, 35 %) Found: MH^+, 962.4866. $C_{52}H_{69}N_7O_6S_2$11B requires 962.4844; Δ = 2.2ppm).

Spectra for receptor (11).

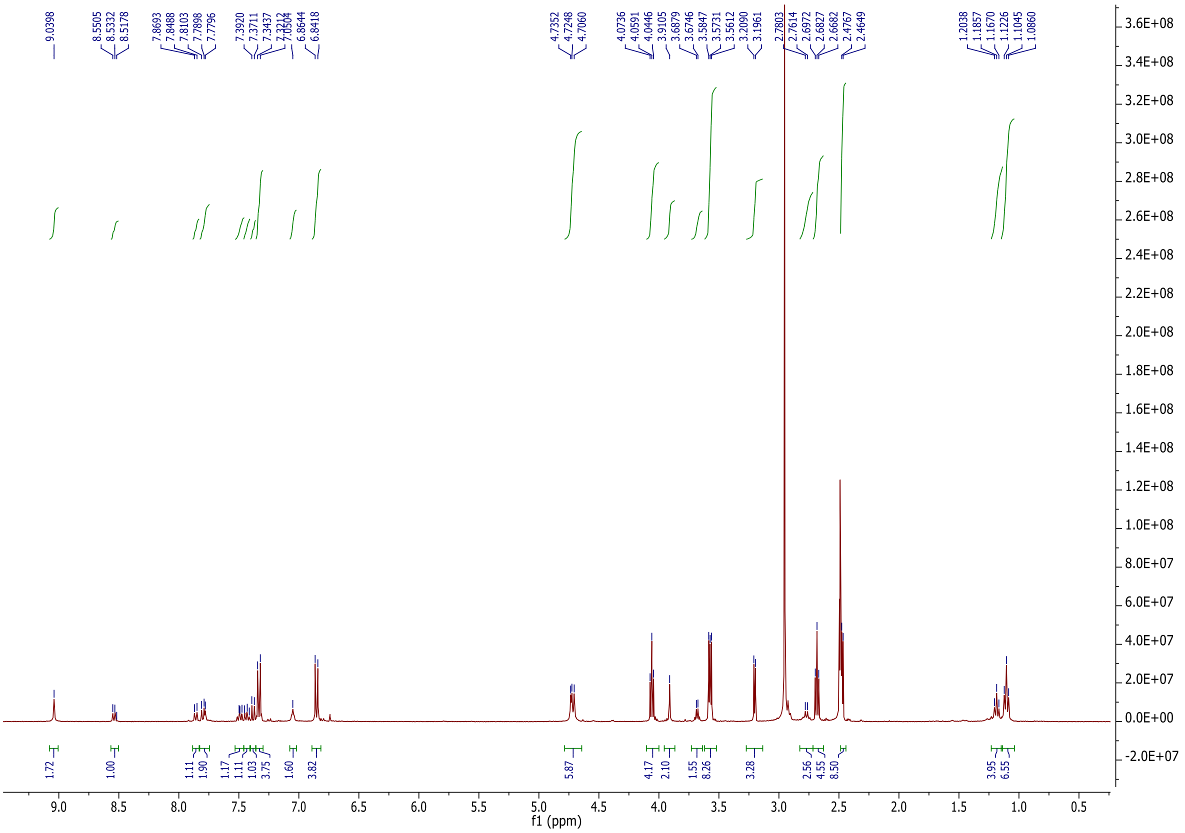

Figure S29. ^{1}H NMR spectrum of receptor (11).

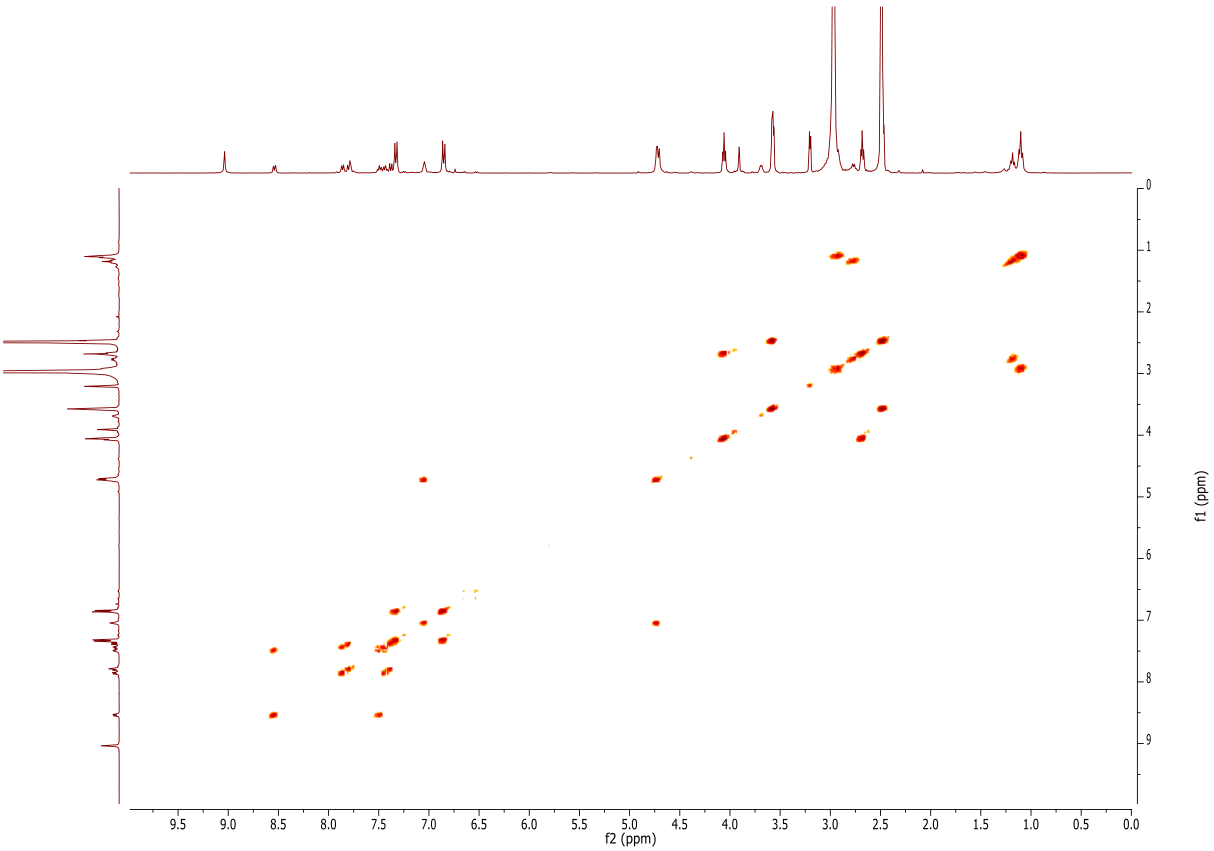

Figure S30. COSY spectrum of receptor (11).

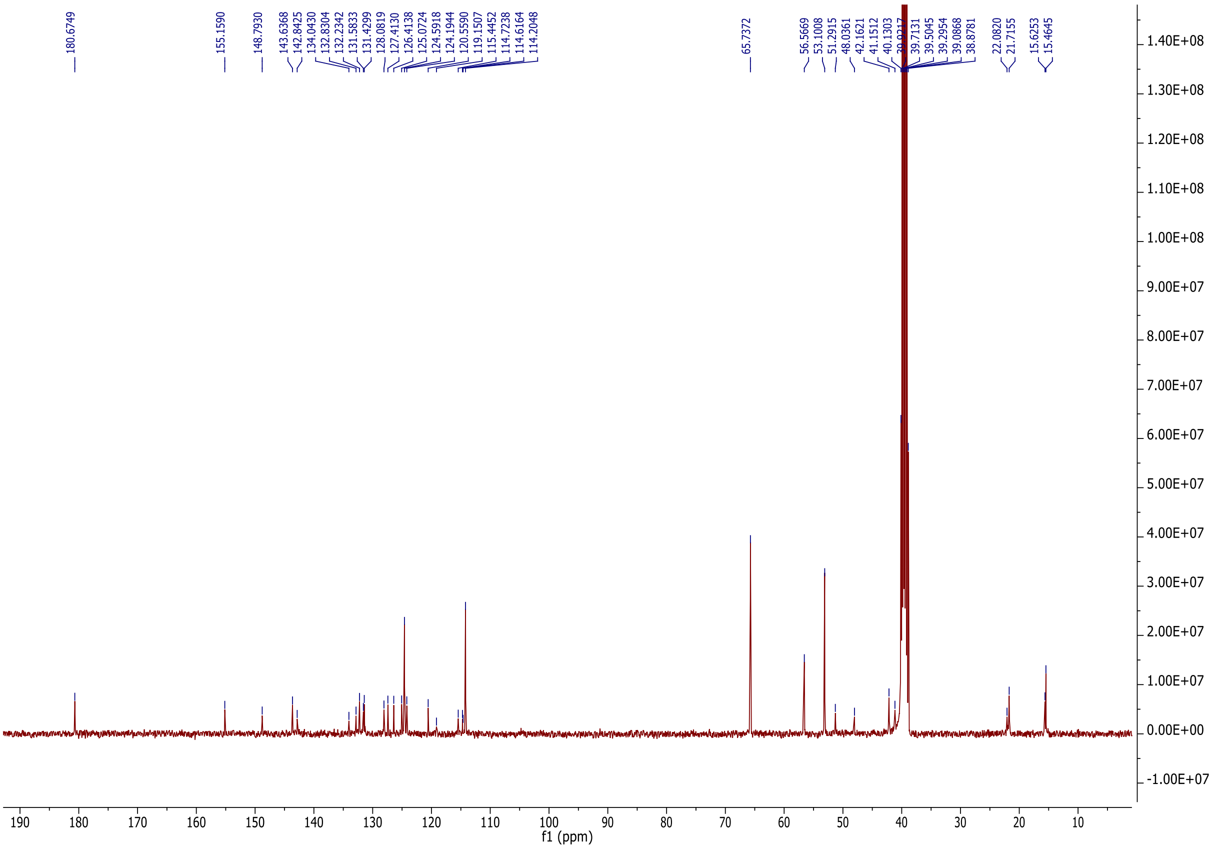

Figure S31. ^{13}C NMR spectrum of receptor (11).

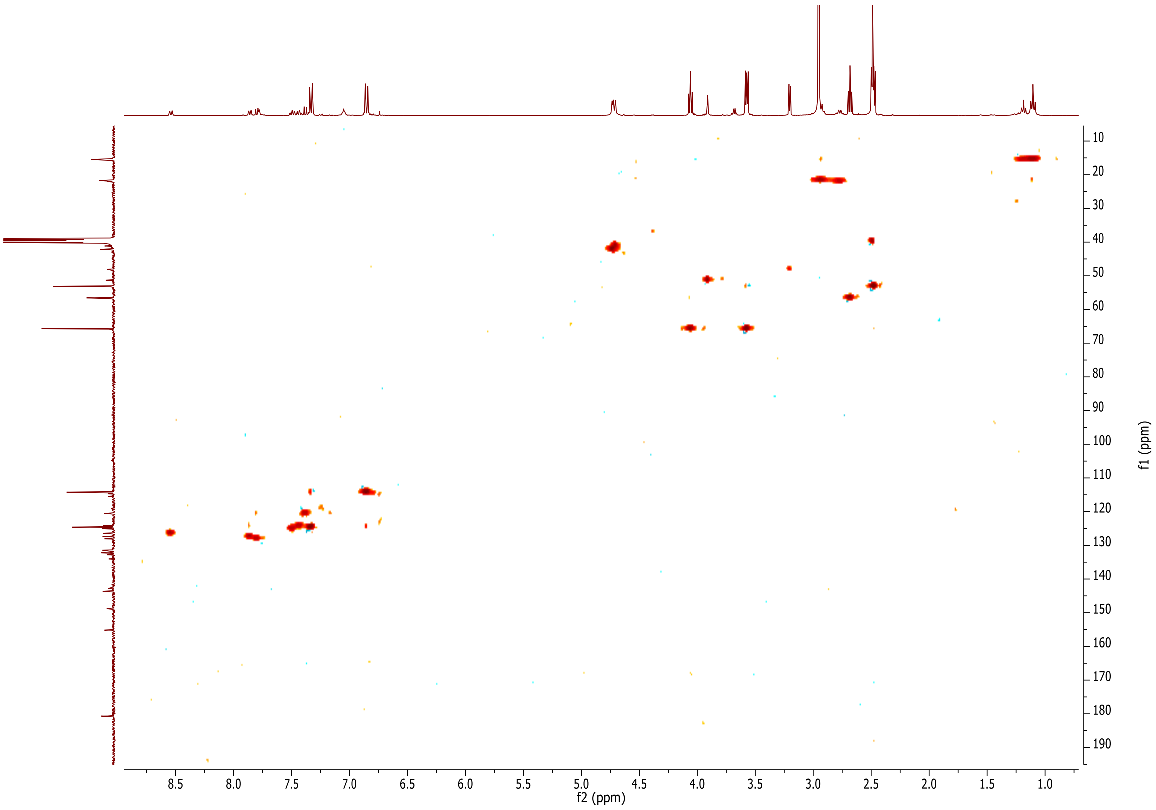

Figure S32. HSQC spectrum of receptor (11).

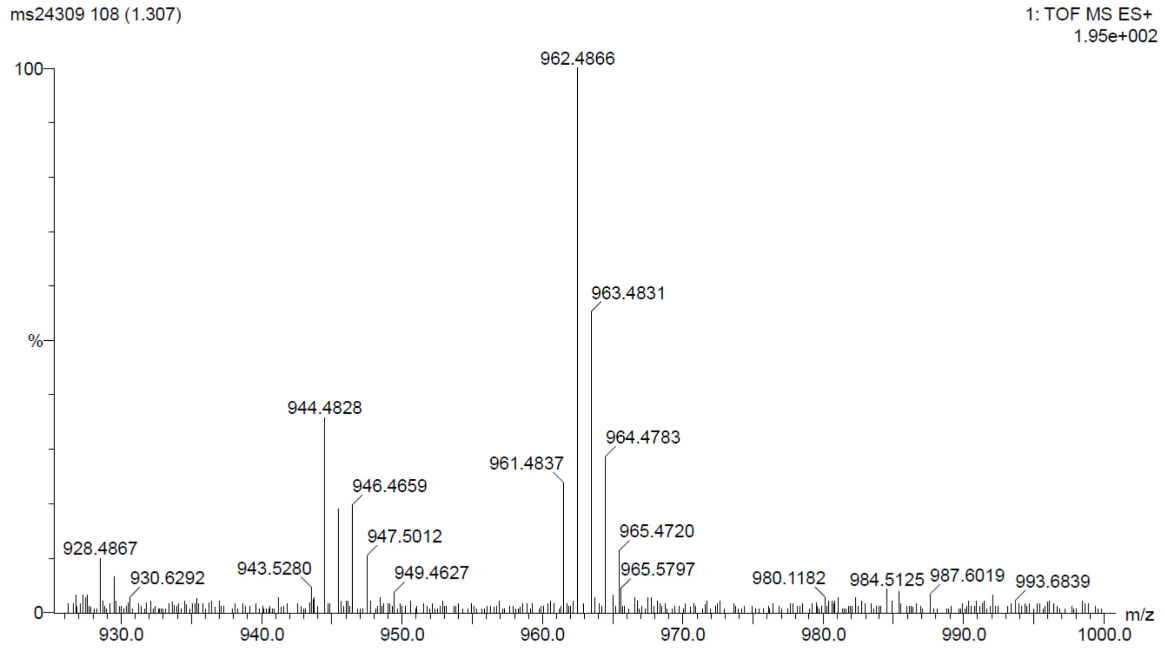

	Mass	Calc. Mass	mDa	PPM	DBE	i-FIT	i-FIT (Norm)	Formula
Minimum:					-1.5			
Maximum:			5.0	8.0	50.0			
	962.4866	962.4844	2.2	2.3	22.5	20.9	0.4	C52 H69 N7 O6 S2 11B

Figure S33. MS spectrum of receptor (11).

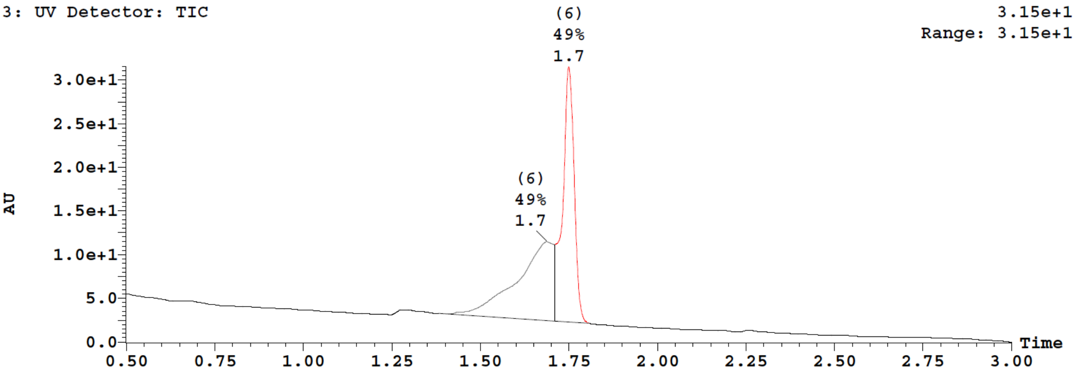

Peak Number	Compound	Time	AreaAbs	Area %Total	Width	Height	Mass Found
6		1.69	1e+006	48.65	0	9e+006	
7		1.75	1e+006	51.35	0	3e+007	

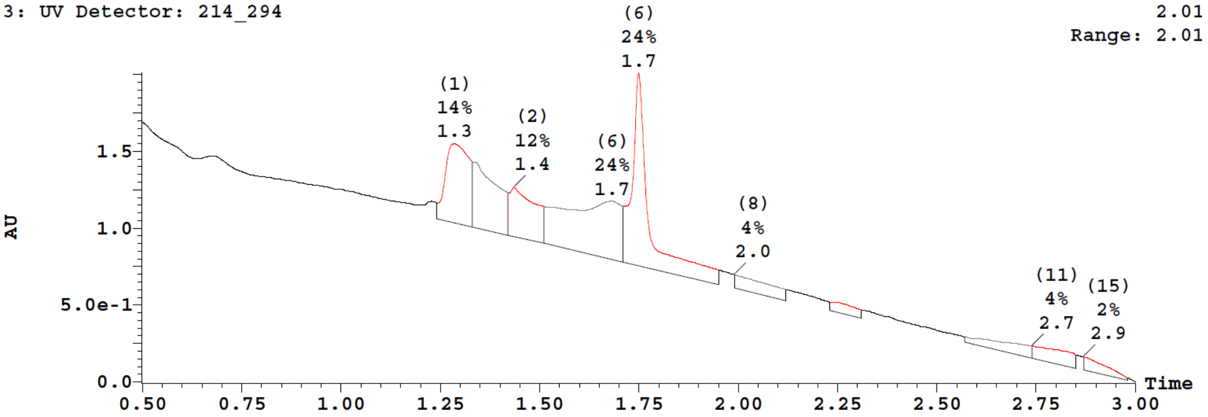

Peak Number	Compound	Time	AreaAbs	Area %Total	Width	Height	Mass Found
1		1.28	4e+004	14.35	0	5e+005	
2		1.34	3e+004	11.64	0	4e+005	
3		1.44	2e+004	9.16	0	3e+005	
6		1.68	6e+004	23.82	0	4e+005	
7		1.75	6e+004	24.86	0	1e+006	
8		1.99	1e+004	4.14	0	9e+004	
9		2.23	5e+003	1.96	0	6e+004	
11		2.57	1e+004	3.87	0	3e+004	
13		2.74	1e+004	3.75	0	8e+004	
15		2.87	6e+003	2.44	0	8e+004	

Peak ID	Compound	Time	Mass Found
1		1.33	

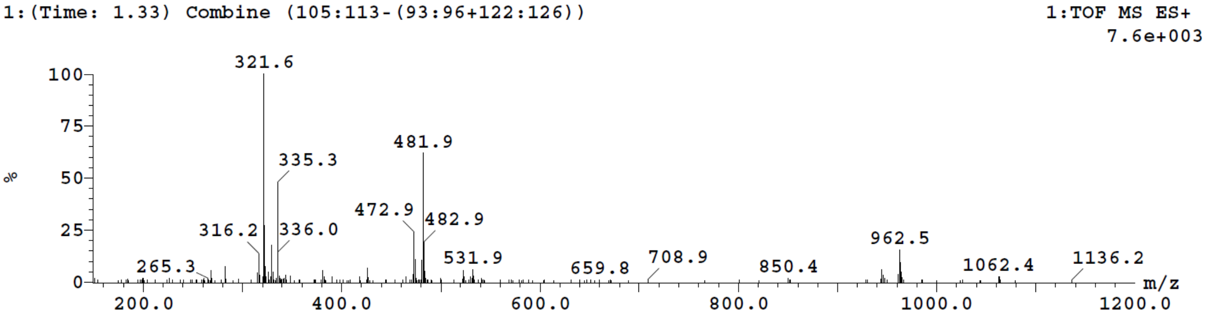

Peak ID	Compound	Time	Mass Found
3		1.48	

Figure S34. LCMS analysis of receptor (11).

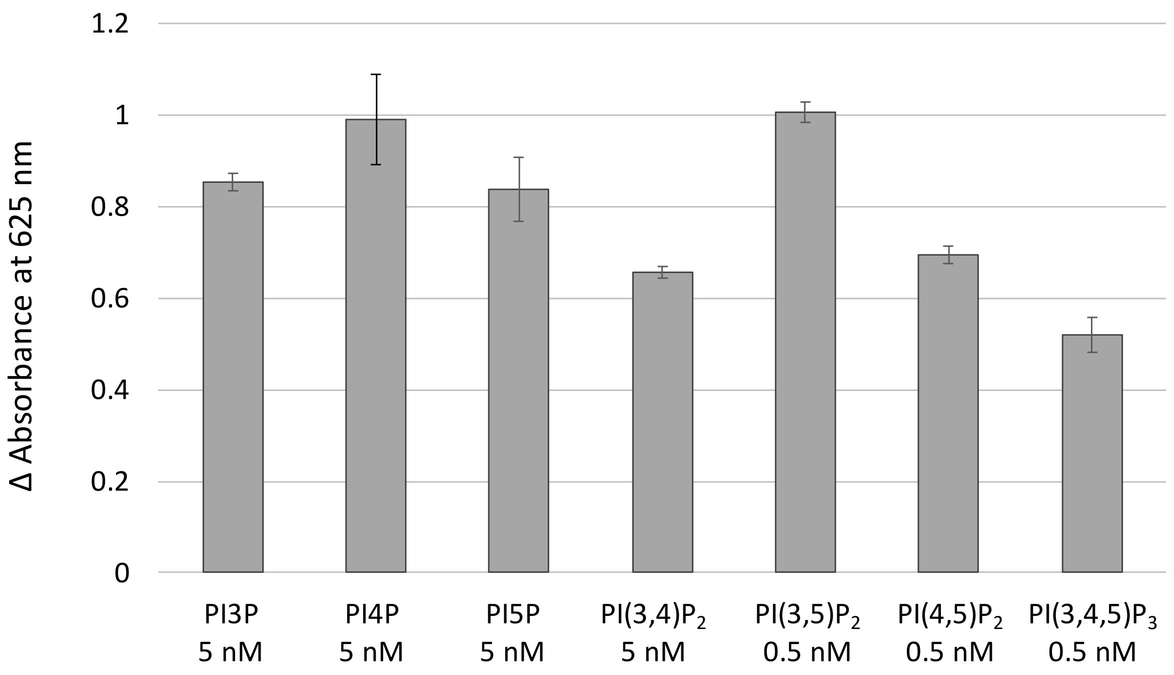

Figure S35. Comparison of the AP activity rates for PIps. Absorbance at 625 nm was recorded using a constant lipid concentration of 40 μM. The alkaline phosphatase concentrations was 5 nM (for PI(3)P, PI(4)P and PI(5)P) and 0.5 nM for PI(3,5)P_2 and PI(4,5)P_2 as sufficient AP activity was recorded at this concentration. Each independent experiment was performed in triplicates. Error bars represent ± standard deviation of one experiment carried out in triplicates (n=3).

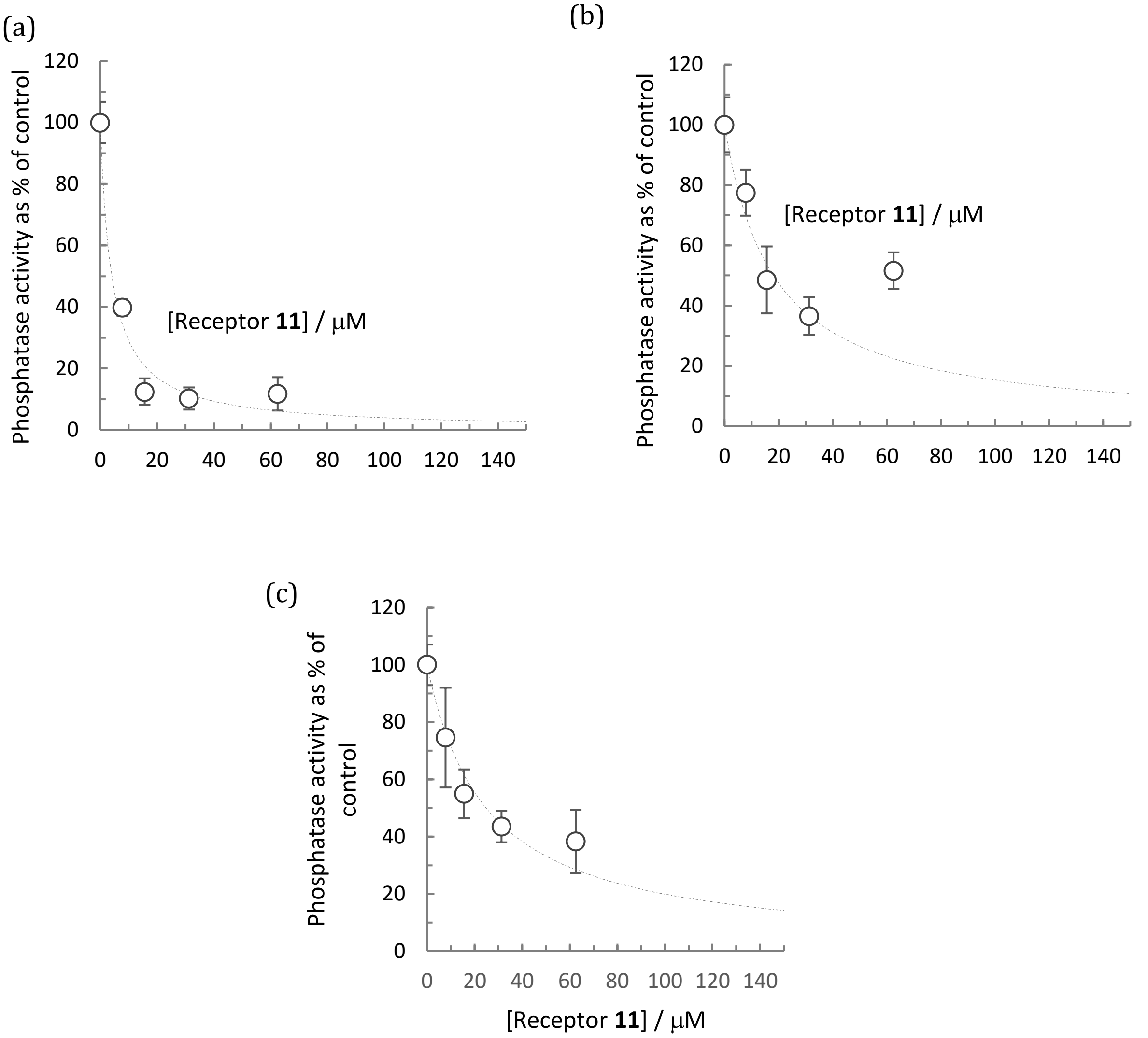

Figure S36. Alkaline phosphatase activity upon incubation of receptor 11 with PI(3)P (a), PI(4)P (b) and PI(5)P (c). Increasing concentrations of receptor **11** were incubated with PI(3)P (a), PI(4)P (b) and PI(5)P (c) (40 μM) for 1 hour before phosphatase (5 nM) was added and the enzyme reaction was carried out at 37 °C for 30 min before it was stopped by addition of phosphate detection reagent. The absorbance is plotted as a percentage of control where no receptor is present (no receptor set at 100 %). Data represented are the mean of two independent experiments performed in triplicates. Error bars represent ± standard deviation of two independent repeats carried out in triplicates (n=6). The dashed line represents fitting performed in MatLab, R^2 = 0.7821 for (a), 0.9078 for (b) and 0.8171 for (c). For PI(4)P (b) the last data point was excluded from the fitting.

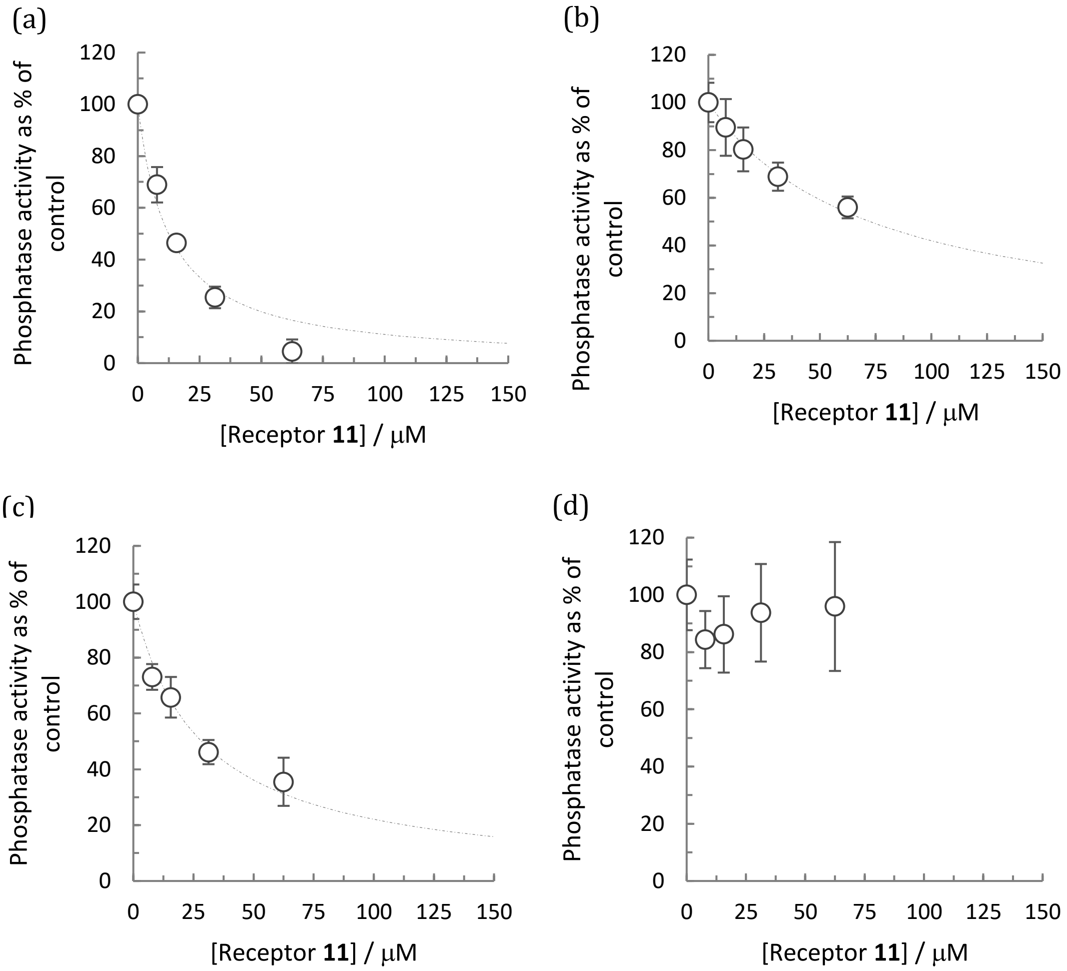

Figure S37. Alkaline phosphatase activity upon incubation of receptor 11 with PI(3,4)P$_2$ (a), PI(3,5)P$_2$ (b), PI(4,5)P$_2$ (c) and PI(3,4,5)P$_3$ (d). Increasing concentrations of receptor **11** were incubated with PI(3,4)P$_2$ (a), PI(3,5)P$_2$ (b), PI(4,5)P$_2$ (c) and PI(3,4,5)P$_3$ (d) (40 μM) for 1 hour before phosphatase was added and the enzyme reaction was carried out at 37 °C for 30 min before it was stopped by addition of phosphate detection reagent. The absorbance is plotted as a percentage of control where no receptor is present (no receptor set at 100 %). Data represented are the mean of two independent experiments performed in triplicates. Error bars represent ± standard deviation of two independent repeats carried out in triplicates (n=6). The dashed line represents fitting performed in MatLab, R^2 = 0.9059 for (a), 0.9834 for (b) and 0.9436 for (c).

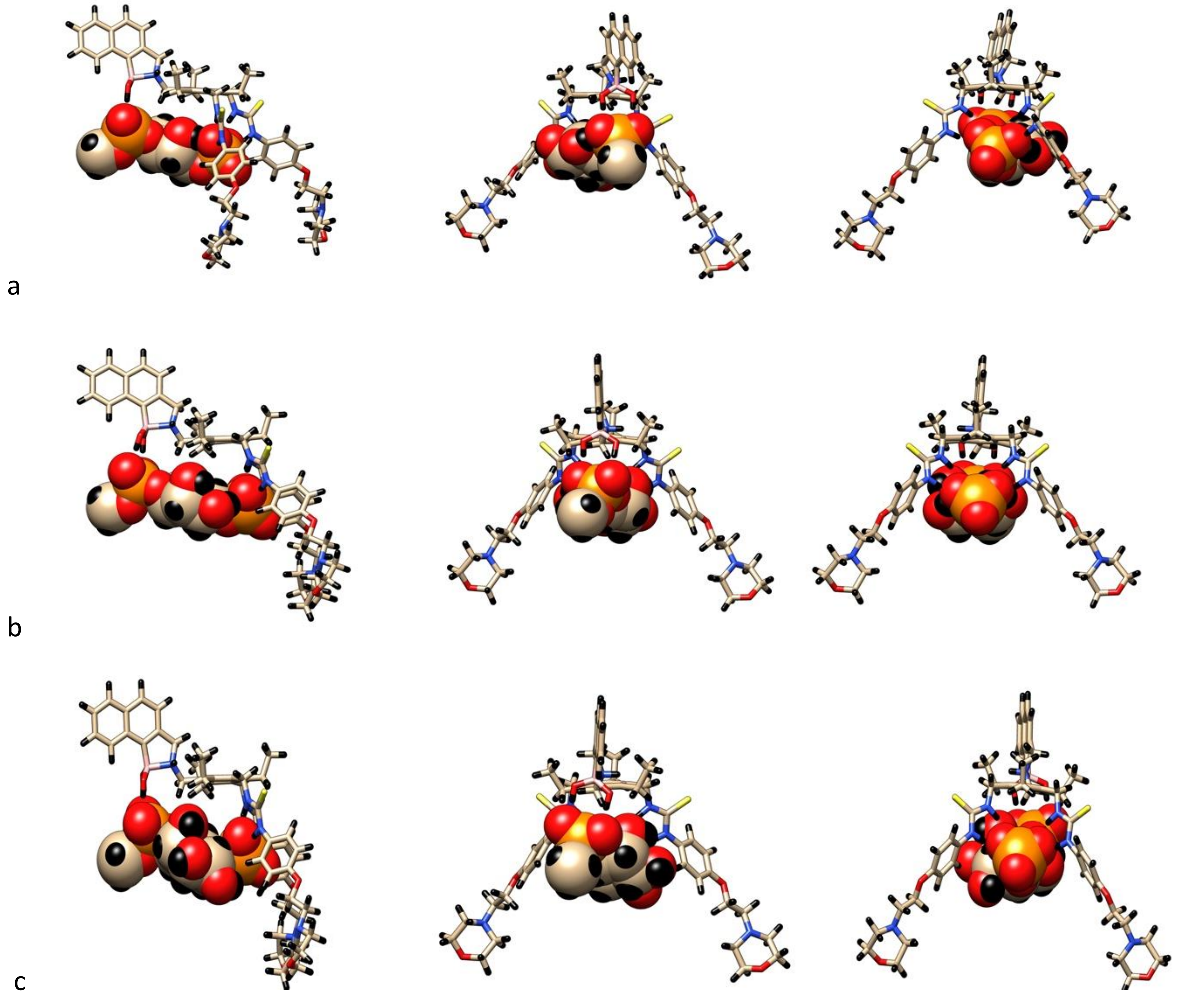

Figure S38. Fully optimised structures of the mono-phosphorylated PIP's with receptor **11** – side, head-on and from behind views left to right respectively; (a) PI(3)P, (b) PI(4)P and (c) PI(5)P bound.

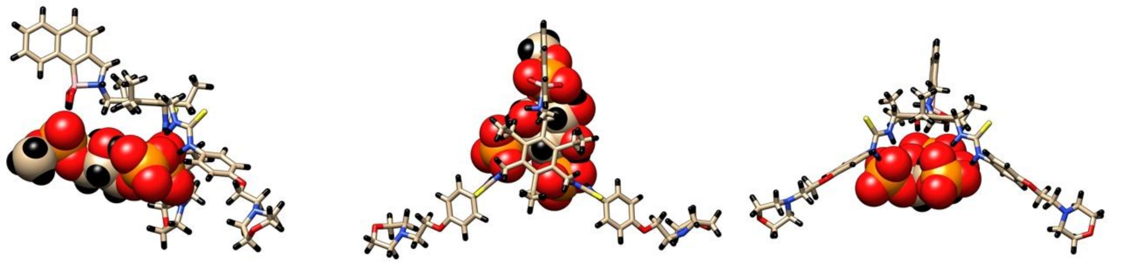

a

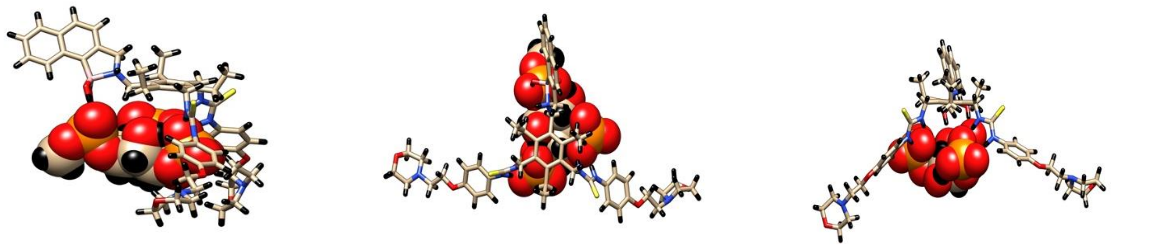

b

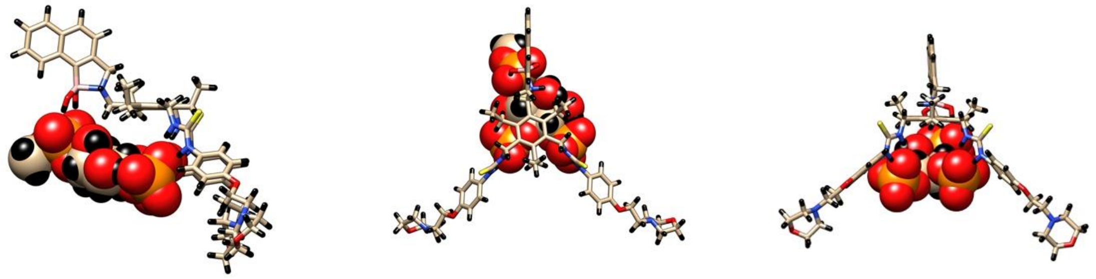

c

Figure S39. Fully optimised structures of the bis-phosphorylated PIP's with receptor **11** – side, head-on and from behind views left to right respectively; (a) PI(3,4)P_2, (b) PI(3,5)P_2 and (c) PI(4,5)P_2

Table S1. Hydrogen-bond distances and "bite" angle for the host-guest complexes between mono- and bi-phosphorylated PIPs and receptor **11**. Distances are in Ångstroms and the angle is in degrees.

Complex	H-Bond H1-01P	H-Bond H2-O2P	H-Bond HA-O3P	H-Bond HC-O3P	H-Bond HB-O5P	H-Bond HD-O5P	Angle NC-C1-ND
PI(3)P	1.873	1.877	1.896	1.890	2.081	1.784	88.78
PI(4)P	1.85	1.925	2.024	1.791	1.859	1.872	88.25
PI(5)P	1.826	1.882	1.878	1.797	1.860	1.802	90.02
PI(3,4)P_2	1.802	1.877	1.889	1.760	1.800	1.721	106.35
	H1-O1P	H2-O2P	HA-O5	HC-O3	HB-O8	HD-O8	NC-C1-ND
PI(3,5)P_2	1.776	1.932	1.756	1.747	1.932	1.694	110.07
	H1-O1P	H2-O2P	HA-O5	HC-O5	HB-O7	HD-07	NC-C1-ND
PI(4,5)P_2	1.873	1.778	1.937	1.743	1.920	1.730	102.07
